# GRB2 is a BECN1 interacting protein that regulates autophagy

**DOI:** 10.1038/s41419-023-06387-7

**Published:** 2024-01-05

**Authors:** Jetsy Montero-Vergara, Kira Plachetta, Lisa Kinch, Stephan Bernhardt, Kriti Kashyap, Beth Levine, Lipi Thukral, Martina Vetter, Christoph Thomssen, Stefan Wiemann, Samuel Peña-Llopis, Verena Jendrossek, Silvia Vega-Rubin-de-Celis

**Affiliations:** 1 Institute of Cell Biology (Cancer Research), University Hospital Essen, Virchowstrasse 173, D-45122 Essen, Germany; 2 University of Texas Southwestern Medical Center, 5323 Harry Hines Blvd., Dallas, TX 75390 USA; 3 Division of Molecular Genome Analysis, German Cancer Research Center (DKFZ), Im Neuenheimer Feld 280, D-69120 Heidelberg, Germany; 4 CSIR-Institute of Genomics and Integrative Biology, Mathura Road, New Delhi Delhi, 110025 India; 5 Academy of Scientific and Innovative Research (AcSIR), Ghaziabad, 201002 India; 6 Department of Gynaecology, Martin Luther University Halle-Wittenberg, Ernst-Grube-Str. 40, D-06120 Halle (Saale), Germany; 7 Translational Genomics. Department of Ophthalmology, University Hospital Essen, Essen, Germany; 8 German Cancer Consortium (DKTK) and German Cancer Research Center (DKFZ), Heidelberg, Germany

**Keywords:** Macroautophagy, Breast cancer

## Abstract

GRB2 is an adaptor protein of HER2 (and several other tyrosine kinases), which we identified as a novel BECN1 (Beclin 1) interacting partner. GRB2 co-immunoprecipitated with BECN1 in several breast cancer cell lines and regulates autophagy through a mechanism involving the modulation of the class III PI3Kinase VPS34 activity. *In ovo* studies in a CAM (Chicken Chorioallantoic Membrane) model indicated that *GRB2* knockdown, as well as overexpression of GRB2 loss-of-function mutants (Y52A and S86A-R88A) compromised tumor growth. These differences in tumor growth correlated with differential autophagy activity, indicating that autophagy effects might be related to the effects on tumorigenesis. Our data highlight a novel function of GRB2 as a BECN1 binding protein and a regulator of autophagy.

## Introduction

Breast cancer is the leading cause of cancer-related death in women worldwide [1]. Around 15–20% of those cancers have amplifications in HER2, a receptor tyrosine kinase (RTK) of the EGFR family [2]. Although there are multiple therapies available, many patients relapse or develop resistances and then have a poor prognosis [3]. We previously showed that BECN1 and HER2 interact in multiple HER2 overexpressing breast cancer cell lines and xenografts, and that HER2 overexpression inhibits autophagy [4]. In addition, we found that HER2 mutants inhibit autophagy through BECN1 phosphorylation, similarly to other tyrosine kinases [5]. Interestingly, wild-type HER2 does not appear to phosphorylate BECN1, and inhibits autophagy independent of BECN1 phosphorylation in an mTORC1-dependent manner. Thus, we hypothesized that other proteins might mediate the effect of HER2 on BECN1, and on autophagy.

Here, we have identified GRB2 (Growth factor receptor-bound protein 2), an adaptor protein of HER2, as a potential BECN1 interacting partner, based on data derived from a large-scale screen of the autophagy proteins network [6]. Previous studies suggested that GRB2 might be involved in breast tumorigenesis, since mice overexpressing *Grb2* enhanced mammary tumorigenesis [7], while *Grb2*^+/-^ mice delayed the onset of mammary tumors [8]. GRB2 is a ubiquitously expressed adaptor protein that is essential for several cellular functions including cell growth, development, proliferation and metabolism [9, 10]. These phenotypes are associated with its critical function in several oncogenic signaling pathways, such as the PI3K/AKT, SHP2/ERK and JAK/STAT, EGFR, MAPK and RAS pathways [11, 12]. There, GRB2 binds to activated RTKs and then recruits downstream signaling proteins with a single Src homology 2 (SH2) and two flanking SH3 domains, for activation of downstream pathways [13].

The initial goal of the project was to determine the role of GRB2 in the inhibition of autophagy via the HER2 and BECN1 axis, as well as its implications in tumor growth. However, we found that GRB2 indeed regulates autophagy not only in HER2-overexpressed cells but also independently of HER2, suggesting that GRB2 is a major autophagy regulator.

## Results and discussion

### GRB2 and BECN1 co-immunoprecipitate in multiple cell lines

**Fig. 1:**
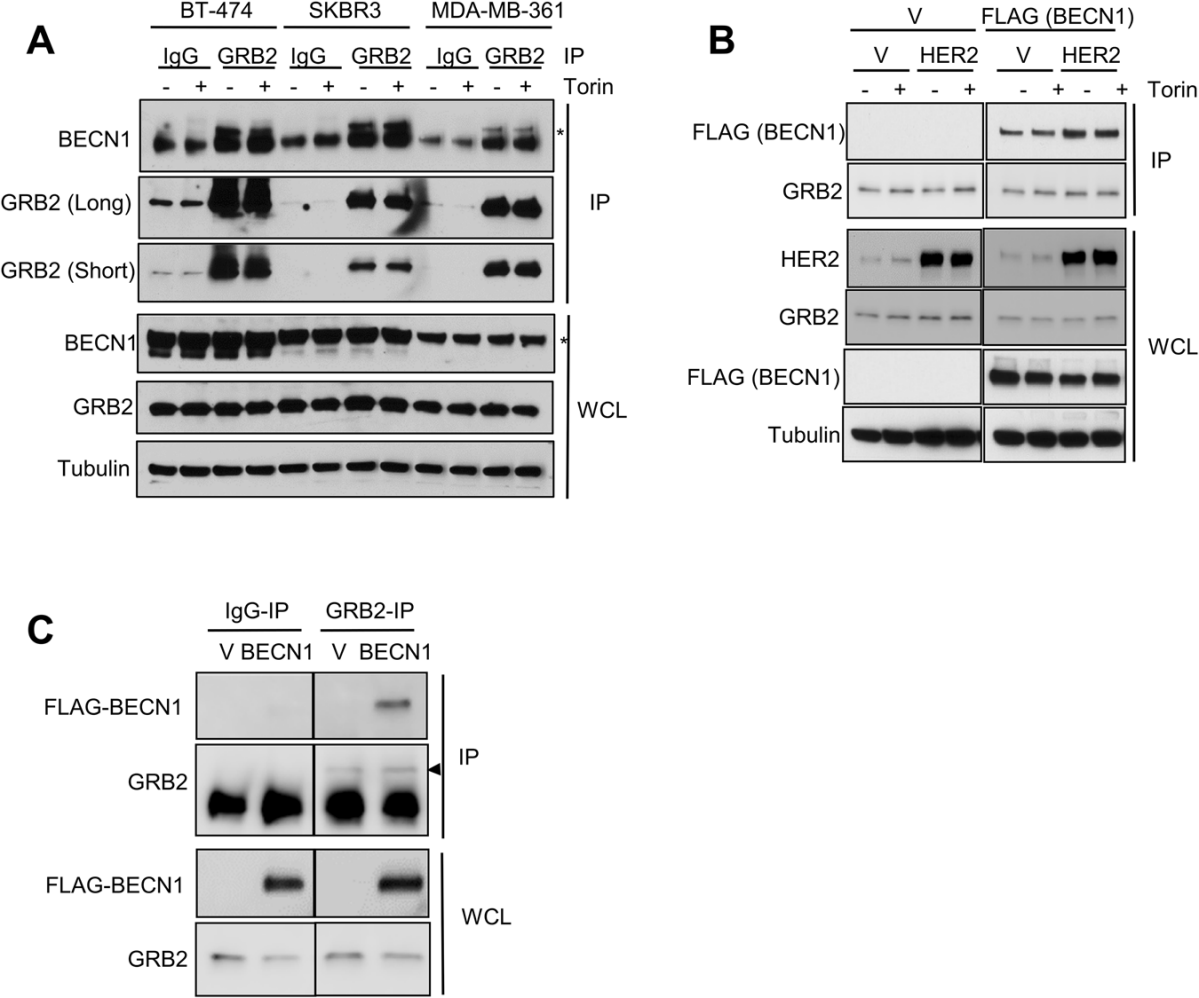
GRB2 and BECN1 co-immunoprecipitate in multiple cell lines. GRB2 was immunoprecipitated from different cell lines followed by western blotting analysis: **A** BT-474, SKBR3 and MDA-MB-361 cells were treated with Torin (0.25 μM for 3 h, or DMSO control). Asterisk indicates the band for BECN1, the bottom band on the IP panel corresponds to the IgG heavy chain; **B** MCF7 cells overexpressing the indicated plasmids and treated with Torin 1 (or DMSO control; 3 h, 0.25 μM); **C** HeLa cells overexpressing FLAG-BECN1 (BECN1) or an empty vector control (V), the arrow indicates the band for GRB2, the bottom band on the IP panel corresponds to the IgG light chain. IP: immunoprecipitation; IgG: IgG control; V: Vector control; WCL: whole cell lysate.

A search for BECN1-interacting proteins into a network database of the autophagy system revealed that GRB2 may bind to BECN1 in an mTORC1-dependent manner. To validate this, GRB2 was immunoprecipitated from three breast cancer cell lines expressing high levels of HER2 (i.e., SKBR3, BT474 and MDA-MB-361) in absence and presence of Torin 1, an mTOR inhibitor. Endogenous BECN1 co-immunoprecipitated with endogenous GRB2 in all cell lines tested, although independently of mTOR activity since mTOR inhibition did not affect the binding in this context (Fig. 1A, Supplementary Fig. S1A).

GRB2 is the adapter protein that connects phosphorylated RTKs/HER with downstream signaling pathways [14]. GRB2 can also activate RAS with membraneless protein granules (which seem to involve mostly ALK and RET receptor tyrosine kinases), being also able to function independent of HER2 or other members of the HER family [15]. Therefore, we asked whether the BECN1-GRB2 interaction occurred only in the context of HER2 overexpression/amplification or it is a broader event. To test this, HER2-negative breast cancer cells (MCF7) that are estrogen and progesterone receptor positive and express low levels of BECN1 [16], were transduced to stably express HER2 and FLAG-tagged BECN1 (or the corresponding empty vector controls). Endogenous GRB2 was immunoprecipitated after Torin 1 treatment, and analyzed by western blotting. FLAG-BECN1 immunoprecipitated with GRB2 in all samples from cells overexpressing HER2, as well as in empty vector control cells, suggesting that this interaction is at least partially independent of HER2 (Fig. 1B). In addition, more FLAG-BECN1 co-immunoprecipitated with GRB2 when HER2 was overexpressed, suggesting that even though HER2 is not required for GRB2/BECN1 binding, HER2 may modulate their interaction.

Since the BECN1/GRB2 interaction seemed to be partially independent of HER2 in breast cancer cell lines, we next tested if this interaction also occurred in other, not breast cancer, cell lines, such as HeLas (cervical adenocarcinoma). In line with the observations described above, GRB2 co-immunoprecipitated with FLAG-BECN1 only in lysates from cells overexpressing FLAG-BECN1, whereas there was no interaction detected on the IgG controls or in the GRB2 IP of cells transfected with an empty vector (Fig. 1C). Similar results were obtained when using HEK293FT cells, derived from human embryonal kidney and stably expressing the SV40 large T antigen (Supplementary Fig. 1B). GRB2 was immunoprecipitated from cells depleted of GRB2 by siRNA (or a siRNA control), and a significantly lower amount of FLAG-BECN1 was recovered from the samples that were depleted of GRB2 (Supplementary Fig. S1B).

Taken together, our data indicate that GRB2 and BECN1 interact also independently of HER2 and mTOR in multiple cell lines, suggesting that such binding could be a general event that takes place in all cells.

### GRB2 regulates autophagy

**Fig. 2:**
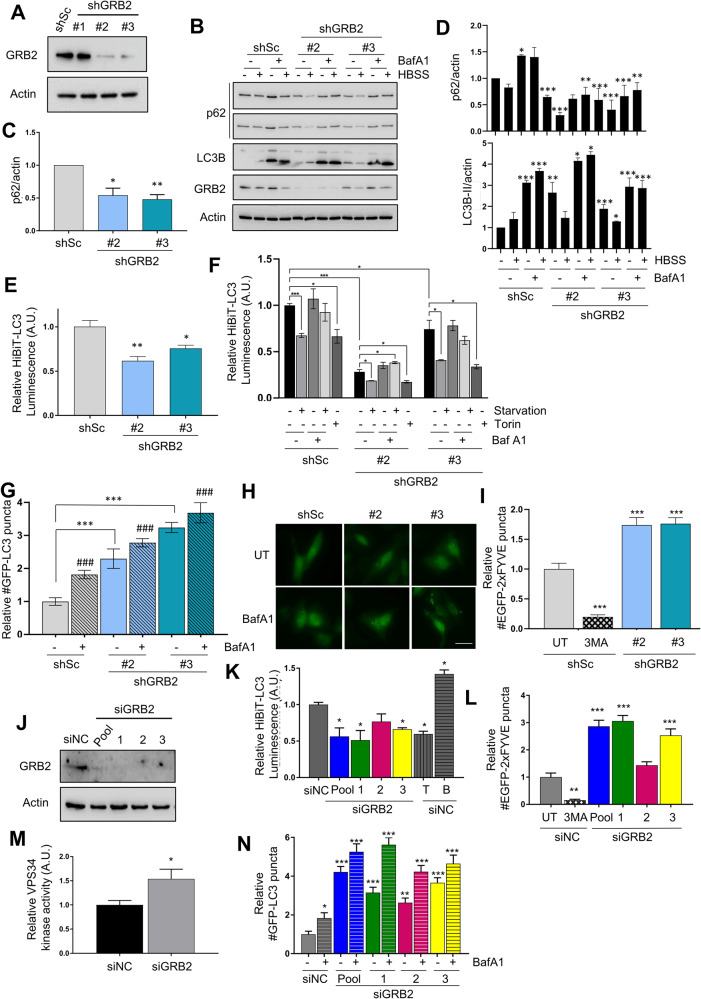
GRB2 regulates autophagy. **A** *GRB2* knockdown efficiency in HeLa cells transduced with the indicated shRNAs was analyzed by WB. Autophagic flux on these cells was analyzed by: **B** Western blot analysis of p62 and LC3B of cells incubated in normal or starvation media (HBSS) for 3 h, with or without Bafilomycin A1 (BafA1 100 nM, 3 h), quantification of p62 basal levels of three independent experiments (**C**) and p62 and LC3B-II of all conditions (**D**) is shown. ***, *P* < 0.001; **, *P* < 0.01; *, *P* < 0.05; *t*-test comparing each sample to un*t*reated shSc control; HiBiT-LC3 luminescent assay of basal levels of autophagy (**E**) and in response to starvation (HBSS) or Torin (**F**) (*n* = 3); **G**, **H** GFP-LC3 puncta numbers at basal levels or treated with Bafilomycin A1 (100 nM, 3 h, *n* = 3; # indicates comparisons of bafilomycin-treated *vs*. untreated for each condition with a value of ^###^ *P* < 0.001); (**I**) PI3P formation in cells treated or not with 3-MA (2.5 mM for 3 h, *n* = 3) as control. HeLa cells depleted of GRB2 using three different siRNA oligos individually or as a pool were analyzed by WB (**J**), HiBiT-LC3 luminescence assay (*n* = 3) (**K**), EGFP-2xFYVE puncta (*n* = 3) (**L**), VPS34 in vitro kinase activity (**M**), and GFP-LC3 autophagosome formation assay (*n* = 3) (**N**). ShSc: scrambled control shRNA; sh1, sh2, sh3: shRNA targeting *GRB2* with different shRNA sequences. V: empty vector, T: Torin, B: Bafilomycin A1. Data are presented as mean ± S.E.M. A minimum of 50 cells per condition were counted. ***P < 0.001; **P < 0.01; *P < 0.05; one-way ANOVA test.

Having established that GRB2 binds to BECN1, an essential protein for autophagy, we next wanted to analyze if GRB2 had an effect on autophagy. To this end, we employed HeLa cells expressing short hairpin RNAs (shRNA) to stably knockdown *GRB2* (or a scrambled control), using three different shRNA sequences targeting GRB2. Protein levels were analyzed, and cells with the highest knockdown efficiency (shRNA#2 and shRNA#3; Fig. 2A) were selected for further analysis. Autophagic flux was then analyzed using several technical approaches: 1) Western blot analysis indicated that the levels of the autophagic substrate p62 were decreased on the cells expressing the shRNA targeting *GRB2*, while the lipidated form of LC3B was increased upon *GRB2* knockdown compared to the control cells (Fig. 2B). Levels of p62/actin were quantified in basal conditions (Fig. 2C), as well as LC3B-II/actin in cells exposed to starvation (a major autophagy inducer) and bafilomycin A1 (an autophagy inhibitor) (Fig. 2D). 2) Cells were analyzed using a HiBiT-LC3 reporter [17], which enables the detection of the total levels of a HiBiT (luciferase subunit)-tagged LC3 protein. Lysates of cells containing the Large BiT (LgBiT: inactive luciferase subunit), generates an active luciferase enzyme upon binding to HiBiT that produces light after addition of the luciferase substrate and correlates with the total LC3B levels. Depletion of GRB2 led to a significant induction of autophagy compared to the control cells as assessed by this assay in basal conditions (Fig. 2E). In addition, shGRB2 cells significantly induced autophagy both upon starvation as well as with Torin treatment. Interestingly, the decreased luminescence observed in this assay with the shRNAs is at a similar extent as the shSc control treated with Torin (Fig. 2F). 3) Autophagy flux was further analyzed through quantification of autophagosome formation using a GFP-LC3 reporter. There, an increment of autophagosome numbers upon *GRB2* knockdown compared to control (Fig. 2G, H) was found both in basal conditions as well as upon bafilomycin A1 treatment, indicating that this increase was due to an induction of the autophagic flux rather than a block in autophagosome maturation. Data obtained in all these approaches supported our hypothesis that GBR2 is indeed an effector of autophagy.

To further explore the mechanism whereby GRB2 regulates autophagy, we next analyzed whether the activity of VPS34, the catalytic subunit of the Class III PI3Kinase complex 1 (PI3KC3-C1), was modulated by GRB2. For this purpose, VPS34 catalytic activity was indirectly measured through a EGFP-2xFYVE fluorescent reporter in which PI3P (Phosphatidylinositol-3-phosphate) formation at the autophagosomes was observed through the binding of the FYVE domain to PI3P, a phospholipid that is a precursor of the pre-autophagosomal membrane and is produced by VPS34 [18]. HeLa cells depleted of GRB2 through stable *GRB2* shRNA knockdown were transfected with a plasmid encoding for EGFP-2xFYVE, and samples treated with the VPS34 inhibitor 3-MA were used as negative controls. *GRB2* knockdown increased the number of EGFP-2xFYVE dots compared to the control cells (Fig. 2I), which correlated with the induction of autophagy we had determined (Fig. 2B–H). Indeed, VPS34 also co-immunoprecipitated with GRB2 in multiple cell lines (Supplementary Fig. S1C–E).

Because GRB2 is an essential protein for cells to survive, cells stably expressing shRNAs targeting GRB2 with the strongest knockdown did not survive, whereas the cells that still expressed GRB2 were able to survive and proliferate therefore taking over all of the population. Thus, to minimize this effect and with the purpose of having as little GRB2 expressed in the cells as possible, the above-mentioned experiments were repeated in HeLa cells with transient and acute *GRB2* knockdown using three different oligos as well as a pool of all of them (Fig. 2J). Analysis on the HiBiT-LC3 assay demonstrated that all oligos significantly decreased luminescence, having an effect similar to the cells treated with Torin, whereas treatment with Bafilomycin A1 increased the readings, as expected for an autophagy inhibitor (Fig. 2K). Furthermore, the number of EGFP-2xFYVE dots were significantly induced when GRB2 was depleted with two different oligos as well as with the pool, whereas only a slight, non-significant increment was detected with another one. This effect on the VPS34 activity was further confirmed using a lipid in vitro kinase assay on samples depleted of GRB2, and a significant increase in its kinase activity was detected (Fig. 2M). Lastly, GFP-LC3 autophagosome formation assay also showed a significant increase in dots when GRB2 was depleted with different oligos that was further increased with Bafilomycin A1 treatment (Fig. 2N).

Taken together these data show that GRB2 is a modulator of autophagy and that its depletion leads to activation of autophagy through modulating the activity of VPS34. Moreover, we obtained similar results in multiple cell lines, including U2OS (derived from osteosarcoma) as well as in HER2-overexpressing breast cancer cell lines BT474 and SKBR3, as follows: Transient siGRB2 knockdown in U2OS cells (Supplementary Fig. S2A–D) showed that GRB2 depletion induces autophagy as assessed by the HiBiT-LC3 assay using three different oligonucleotides, as well as a pool of siRNA (Supplementary Fig. S2B). Positive Torin control also followed the same trend, whereas treatment with Bafilomycin A1 inhibited autophagy as determined by a significant increase in luminescence. In addition, p62 levels decreased as assessed by WB (Supplementary Fig. S2C), as well as the total LC3B levels, indicated an increased autophagic flux and quick turnover of the protein. In addition, EFGP-2xFYVE puncta also increased upon depletion of GRB2 (Supplementary Fig. S2D). Taken together these data also support the hypothesis that *GRB2* knockdown induces autophagy and that it modulates the VPS34 kinase activity in U2OS cells.

Similar experiments were performed in HER2-overexpressing breast cancer cell lines BT474 and SKBR3 depleted of *GRB2* using CRISPR-Cas9 knockout and shRNA or siRNA knockdown, respectively (Supplementary Fig. S2E–L). Two different BT-474 CRISPR-Cas9 clones were analyzed and both of them showed that GRB2 knock-out induced autophagy, as determined by the number of GFP-LC3 puncta (Supplementary Fig. S2F), as well as the VPS34 activity (Supplementary Fig. S2G). SKBR3 cells expressing two different shRNAs targeting *GRB2* (Supplementary Fig. S2H) or several siRNAs (Supplementary Fig. S2K) displayed a significant induction of the autophagic flux measured by the HiBiT-LC3 assay in both systems (Supplementary Fig. S2I, L), and a decrease in p62 levels by WB (Supplementary Fig. S2J). Interestingly, SKBR3 do not strongly induce autophagy in response to starvations (measured by p62 degradation and LC3 lipidation in WB), as previously reported [4], but decreasing the GRB2 levels seem to restore the p62 degradation upon HBSS treatment.

Taken together these results indicate that depletion of GRB2 induces autophagy independently of HER2, and that GRB2 may regulate autophagy through modulating the activity of VPS34.

Due to the implications of GRB2 in the PI3K/AKT/mTOR and JNK/MAPK pathways [10], we next tested if the autophagy effects of GRB2 were dependent on those pathways. To define if the effect of GRB2 was dependent on mTORC1, HeLa cells stably depleted of GRB2 were analyzed for their ability to modulate mTORC1 upon amino acid starvation and subsequent replenishment. This assay had been previously applied to establish the link between mTORC1 activity and amino acid availability [19]. Our results indicated that GRB2 depletion did not affect mTORC1 activity, and mTORC1 was reactivated when re-adding amino acids independently of GRB2 levels, as assessed by phosphorylation of its direct targets RPS6KB1 and EIF4EBP1, as well as RPS6 phosphorylation (Supplementary Fig. S3A). Furthermore, MAPK analysis by WB indicated that GRB2 depletion did not affect the phosphorylation of ERK when comparing to control cells, therefore suggesting that *GRB2* knockdown did not affect the MAPK pathway in our experimental systems (Supplementary Fig. S3B).

### Y52, R86, and S88 are essential for the inhibitory effect of GRB2 on autophagy

**Fig. 3:**
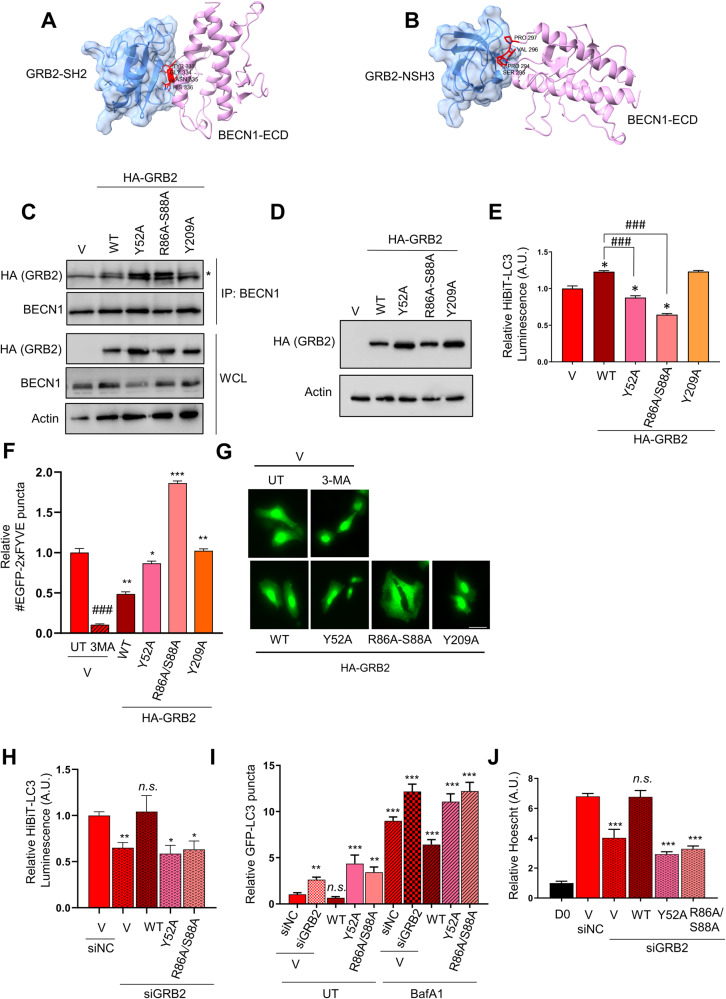
Identification of essential residues of GRB2 for its function in autophagy. Predicted binding modes of BECN1 ECD with GRB2 SH2 domain (**A**) and N-terminal SH3 domain (**B**). SH2 and SH3 consensus binding motifs in BECN1 are colored red. **C** BECN1 immunoprecipitation of stable HeLa cells expressing GRB2 wild type (WT), or the indicated mutants. HeLa cells overexpressing the indicated proteins were analyzed by (**D**) western blot, **E** HiBiT-LC3 luminescence assay (*n* = 5), and (**F, G**) GFP-2xFYVE assay (2.5 mM 3-MA for 3 h), *n* = 3. HeLa cells stably expressing the indicated constructs were transfected with siRNA targeting *GRB2*, siGRB2 (or a non-targeting oligo, siNC) and analyzed by (**H**) HiBiT-LC3 luminescence assay (*n* = 4), **I** numbers of GFP-LC3 puncta (*n* = 3), and (**J**) Hoescht staining (*n* = 3). V: Vector; WCL: whole cell lysate; Asterisk indicates the band corresponding to HA-GRB2 in C. Data are presented as mean ± S.E.M, and a minimum of 50 cells were counted. * compared to V, #, compared to WT. ***, *P* < 0.001; **, *P* < 0.01; *, *P* < 0.05; *n.s*., nonsignificant, one-way ANOVA test.

To better characterize the interaction between BECN1 and GRB2, we next wanted to identify residues in GRB2 that are essential for its binding with BECN1 and required for its inhibitory effect on autophagy. Protein-protein docking studies suggested that GRB2 can indeed interact with BECN1 via diverse interacting surfaces, which resulted in energetically similar binding modes (Fig. 3A, B). In silico modeling of potential interaction sites of BECN1 and GRB2 suggested that the two proteins may directly bind through the BECN1 Evolutionary Conserved Domain (ECD) (Fig. 3A, B). Indeed, our immunoprecipitation experiments in HeLa cells overexpressing FLAG-BECN1-WT or a mutant lacking the ECD domain (dECD, d267–450) indicate that the ECD domain in BECN1 is required for its binding to GRB2 (Supplementary Fig. S4A). The model predicted further that GRB2 residues Y52 in the N-terminal SH3 domain, R86-S88 in the central SH2 domain, and Y209 in the C-terminal SH3 domain were potentially involved in this interaction with BECN1 (Supplementary Fig. S4B, C). Tyrosines Y52 and Y209 in GRB2 have previously been identified to be phosphorylated by EGFR as well as JAK2, and by Bcr/Abl kinases, respectively [20, 21]. Phosphorylation at both sites attenuates MAPK signaling downstream of activated receptors and negatively regulates cell growth. No mutations in Y52 are annotated in the COSMIC database and only one tumor with a mutation in Y209 has been sequenced. Therefore these positions are not considered mutational hot-spots, suggesting that negative regulation of these phosphosites is likely not a driver of disease even though this negatively regulates MAPK signaling.

To test if any of these residues affect binding of GRB2 to BECN1, HeLa cell lines stably overexpressing GRB2-WT or the corresponding non-functional mutants (or an empty vector control) were generated. Immunoprecipitation of endogenous BECN1 followed by WB indicated that the three mutants were still able to bind to BECN1 in comparable amounts to the GRB2-WT (Fig. 3C).

The effect of these mutations in autophagy was also analyzed, and expression levels of the different wild-type and mutant GRB2 proteins were analyzed by western blotting. Even though the levels of the GRB2 mutants were higher than the WT (also taking into account the actin levels) (Fig. 3D), they showed different effects in autophagy compared to the WT or the vector control. For instance, overexpression of the GRB2 WT inhibited autophagy in the HiBiT-LC3 assay, as observed by a significantly higher luminescent signal compared to control. In contrast, proteins containing the mutated residues Y52A and R86A-S88A had lost the ability to inhibit autophagy and rather seemed to act as dominant negative, as HiBiT-LC3 luminescence was significantly decreased as compared both to the empty vector and to the WT controls (Fig. 3E). However, the Y209A mutant had an inhibitory effect similar to the WT (Fig. 3E), suggesting that this residue might not be essential for the inhibitory effect of GRB2 in autophagy.

Furthermore, to determine if this autophagic regulation also occurred through regulating the VPS34 catalytic activity, HeLa cells were transfected with the EGFP-2xFYVE reporter and PI3P formation was measured. Our results indicated that at basal levels, the WT form of GRB2 inhibits VPS34 catalytic activity as expected. In contrast, all mutants lost this effect as shown by increased production of PI3P (Fig. 3F, G), where mutants Y52A and Y209A increased PI3P to similar levels as the empty vector (Fig. 3F, G). However, the Y209A mutant seemed to also lose the activity of the GRB2-WT, whereas it behaved similarly as the WT on other assays of autophagy regulation, suggesting that this residue might be involved in other, autophagy-independent functions of VPS34. Interestingly, the GRB2 mutants tested in this study behaved similarly to GLPR2 (GLI pathogenesis-related 2), a Golgi-associated binding protein of BECN1 that had also been identified as a negative regulator of autophagy through a mechanism involving the regulation of the VPS34 activity [22].

To confirm the differential effects of some of the mutants on autophagy, a GFP-LC3 autophagosome formation assay was carried out and the results indicated that overexpression only of the Y52A and R86A-S88A mutants induced autophagy when compared to the empty vector control (V) or to the WT (Supplementary Fig. 5A, B), supporting the idea that these mutants act as dominant negative. The Bafilomycin A1 control (Supplementary Fig. 5A, B) indicated that the increase in autophagosome formation was due to a higher autophagic flux rather than a block in autophagosome maturation, since higher numbers were also found in these controls. These results confirmed that GRB2 Y52A and R86A-S88A are loss-of-function mutants that are not able to inhibit autophagy.

However, the effects of the GRB2 overexpression (WT or mutants) on autophagy might have been obscured by the endogenous wild-type GRB2 present in the cells. To address this potential issue, cells were transiently transfected with a siRNA oligo targeting the 3’-UTR of *GRB2*, which would only deplete the endogenous GRB2 and not the exogenously expressed GRB2 ORF (Open Reading Frame). To test this system, HeLa cells were transiently transfected with the above-mentioned oligo (siUTR) or with an oligo targeting the GRB2 open-reading frame, which should deplete both, the endogenous *GRB2* and the plasmid-expressed HA-tagged *GRB2* (siORF). A non-targeting control siRNA was included as negative control (siNC), and cells were also transfected with a plasmid overexpressing HA-tagged WT-GRB2 or an empty vector control (Vector). We first validated that only the endogenous and not the GRB2-WT-HA overexpressed protein was targeted with the siUTR oligo, whereas the siORF oligo was able to deplete both GRB2 forms. These tools were then used for multiple experimental approaches (Supplementary Fig. S5C). Indeed, the decrease in luminescence readings detected by GRB2 depletion via siRNA (indicating increased autophagic flux) was rescued by overexpression of WT-GRB2, but not when the loss-of-function mutants were overexpressed (Fig. 3H). In addition, the induction on the autophagosome numbers by *GRB2* knockdown was also rescued by WT-GRB2 overexpression, but not by the loss-of-function mutants Y52A and R86A/S88A (Fig. 3I), and VPS34 activity assessed in vitro confirmed that WT-GRB2 overexpression decreased its activity, whereas the mutants rescued is (Supplementary Fig. S5D). In order to further ascertain the effects of overexpressing GRB2 or the indicated mutants on cell growth and proliferation, cells were plated and analyzed at day 0 or three days after seeding (Fig. 3J). Knockdown of *GRB2* significantly decreased the number of cells by day 3, and GRB2-WT overexpression rescued cell numbers to a similar level as the vector or non-targeting control, but the GRB2 loss-of-function mutants were not able to rescue this effect (Fig. 3K).

In summary, these results indicate that overexpression of GRB2-WT inhibits autophagy, and that Y52, R86 and S88 residues are important for the regulation of autophagy by GRB2.

### GRB2 overexpression induces tumorigenesis in a CAM model

Having shown that *GRB2* knockdown induced autophagy in vitro, we next applied an in ovo model to further assess the effects of GRB2 in tumor formation and tumor growth. To this end, HeLa cells depleted of GRB2 or overexpressing GRB2-WT and the corresponding mutants, respectively were implanted on the chorio-allantoic membrane of fertilized chicken eggs. Here, we focused on the GRB2 Y52A and R86A-S88A mutants as these had shown effects on autophagy in vitro.

*GRB2* knockdown cells were first tested to assess if the transient knockdown of *GRB2* would last until the end of the seven days implantation period (Supplementary Fig. S6A). On the day of implantation, the expression levels of GRB2 were analyzed by western blot to ascertain depletion of GRB2 protein levels (Supplementary Fig. S6B). Results indicated that *GRB2* was crucial for tumor development since only 20% of the eggs in the *GRB2* knockdown group developed a tumor (Supplementary Fig. S6C) compared to the control transfected cells that developed tumors in 85% of the eggs. Besides, the tumors that arose were significantly smaller compared to control in the *GRB2* knockdown group (Supplementary Fig. S6D). Macroscopic observation showed that the GRB2-depleted tumors were less dense and with non-defined borders in comparison to control tumors that had defined borders (Supplementary Fig. S6D). Furthermore, histological analysis indicated that tumors with *GRB2* knockdown had lower p62 levels, suggesting higher autophagy activity (Supplementary Fig. S6E), therefore showing a similar effect to what we had observed in vitro.

**Fig. 4:**
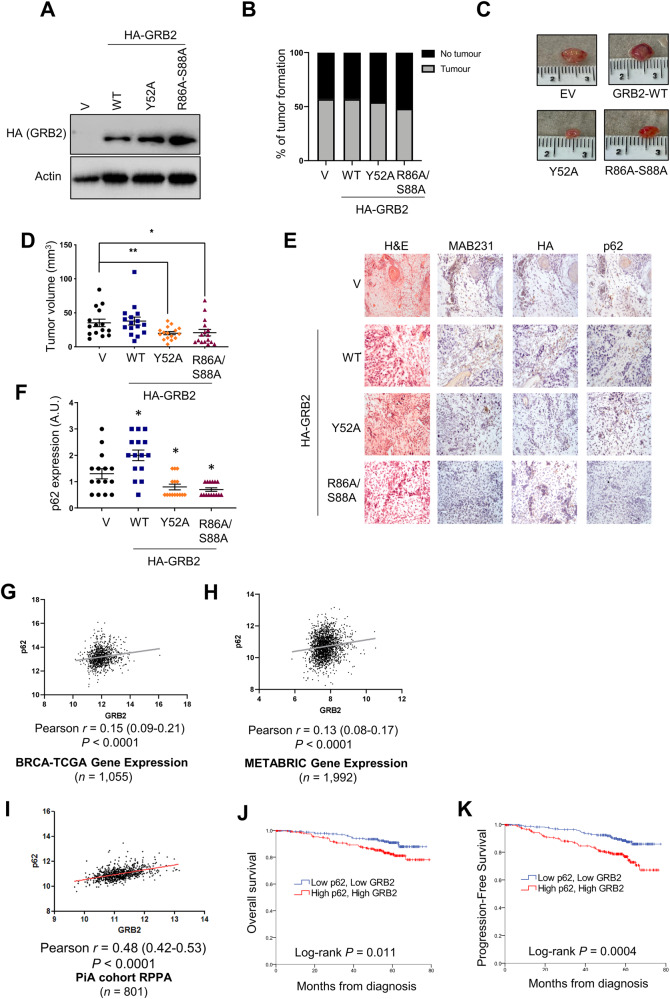
GRB2 loss-of-function mutants decrease tumor growth on a CAM model. **A** WB analysis of HeLa cells stably expressing an empty vector (V), a GRB2 wild-type plasmid (WT), or the indicated mutants on implantation day, **B** percentage of tumors obtained, **C** pictures of extracted tumors, **D** representation of tumor volume. Data are shown as mean ± S.E.M. **, *P* < 0.01; *, *P* < 0.05; *t*-test with Mann-Whitney correction, **E** histological analysis of extracted CAM tumor samples, and p62 quantification (**F**). GRB2 and p62 correlation from gene expression analysis of the BRCA-TCGA (**G**) and METABRIC (**H**) datasets. **I** GRB2 and p62 correlation from RPPA analysis. **J** Overall survival and (**K**) progression free survival of breast cancer patients from the PiA dataset. H&E: hematoxylin eosin, MAB231: nuclei marker.

Since *GRB2* knockdown could likely affect a number of other pathways besides autophagy, we next analyzed the *in ovo* effects in the autophagy loss-of-function mutants. Western blot analysis of cells at the implantation day showed similar expression levels across all the constructs (Fig. 4A), and the percentage of eggs developing tumors was similar in all samples (Fig. 4B). Tumor volume was not significantly different when overexpressing GRB2-WT compared to the vector control. Interestingly, overexpressing both loss-of-function GRB2 mutants were significantly smaller compared to those expressing the vector control and GRB2-WT (Fig. 4C, D). Besides, there were also differences in tumor shape and morphology, with less structured and diffused growth of tumors expressing the GRB2 mutants as compared to the empty vector or GRB2-WT overexpressing tumors. Furthermore, IHC analysis revealed that tumors expressing the GRB2 mutants were associated with an induction of autophagy, as seen by a decreased signal of p62 (Fig. 4E). Quantification of p62 protein indicated a trend towards decreased p62 levels on the tumors overexpressing the GRB2 Y52A and R86A-S88A mutants compared to the GRB2-WT or the empty vector (Fig. 4F).

### GRB2 expression correlates with p62 levels in clinical samples

To determine the potential clinical relevance of our findings, we analyzed gene expression data from the BRCA-TCGA and the METABRIC datasets, and found that RNA expression levels of *GRB2* positively and significantly correlated with the expression of *SQSTM1*/p62 in breast cancer patient samples (Fig. 4G, H). This correlation was even more prominent when tested at the protein level in breast cancer specimens of a prospectively enrolled cohort of 801 early breast cancer patients (PiA cohort [23]), suggesting that patients with low GRB2 may have induced autophagy (Fig. 4I). Furthermore, patients from the PiA cohort with low GRB2 and p62 protein levels presented a better overall and progression-free survival than those with high levels of both proteins (Fig. 4J, K).

In conclusion, we have uncovered that GRB2 mutants Y52A and R86/S88A had lost the ability of GRB2 to inhibit autophagy in vitro and it also compromised tumor growth. Besides, these mutations did not affect the percentages of tumor formation as compared to the vector and WT controls, indicating that they may have little effect in other pathways, especially when compared to the effects on tumor formation of the *GRB2* knockdown. In addition, a correlation between decreased p62 levels and lower GRB2 in patients, also suggest an increased autophagy in patients with low GRB2.

Taken together, our data suggest that GRB2 is a new BECN1 interacting protein and also a potential new regulator of autophagy and tumor growth.

## Materials and Methods

### Cell culture

SKBR3 (Cellosaurus: CVCL_0033), BT-474 (CVCL_0179), MDA-MB-361 (CVCL_0620), MCF-7 (CVCL_0031), and HeLa (CVCL_0030) cell lines were obtained from American Type Culture Collection, cultured in McCoy 5 A, DMEM-F12, RPMI 1640 and DMEM media (Gibco) respectively, and supplemented with 10% fetal bovine serum (FBS; Gibco) and 1% (v/v) penicillin/streptomycin (Gibco). U2OS cells (CVCL_0042) stably transfected with a HiBiT-LC3 reporter system were from Promega, and HEK293FT (CVCL_6911) were from Invitrogen. Cultures were maintained at 37 °C in a humidified atmosphere of 5% carbon dioxide (CO_2_). Cells were sub-cultured using standard procedures, authenticated by STR profiling, and routinely tested for mycoplasma by PCR.

### Antibodies and reagents

Antibodies were used for immunoprecipitation (IP) at a concentration of 1 μg/mg protein and for western blot (WB) at a 1:1000 dilution (unless otherwise specified). They were acquired from the following sources: Santa Cruz: GRB2 (IP, sc-255), BECN1 (IP, sc-48341), BCL2 (WB, and sc-7382-AC for IP), Normal mouse IgG (sc-2025), β-Actin-HRP (sc-8432 HRP); Cell Signaling: GRB2 (WB, 3972 S), ERK1/2 (9102 S), phospho-ERK1/2 (T202/Y204) (9101 S), BECN1 (3738 S), SQSTM1/p62 (D5L7G) (88588 S), PI3 Kinase Class III/ VPS34 (3811 S), S6 Ribosomal Protein (5G10) (2217 S), phospho-S6 Ribosomal Protein (S240/244) (2215 S), p70 S6 Kinase (9202 S), phopsho-p70 S6 Kinase 1 (T389) (9205 S), 4E-BP1 (9452 S), Tubulin (2144 S); Novus Biologicals:LC3B (NB600–148SS); Sigma Aldrich: FLAG M2 (IP and WB, F1365), Biolegend: HA.11 (IP and WB, 901533).

Antibodies for IHC were used at the following concentrations: Biolegend: HA.11 1:1000 (901533); Merck: MAB231-anti nuclei 1:400 (MAB1281); Progen: SQSTM1/p62 (C-terminus) 1:1000 (GP62-C).

Secondary antibodies were obtained from: Cell Signaling: Anti-rabbit IgG HRP-linked (7074 S) and Anti-mouse IgG HRP-linked (7076 S) used at 1:5000 for WB and at 1:500 for IHC.

Reagents were obtained from the following sources: MedChemExpress: Bafilomycin A1 (HY-100558), Invivo Gen: Torin 1 (inh-tor1); Merck: 3-MA (3-MA- CAS 5142–23–4; Calbiochem, 189490–50MG).

Commercial kits were obtained from Promega: Nano-Glo HiBiT Lytic Detection System (N3040).

### Plasmids

EGFP-2x-FYVE was reported elsewhere [22], pBABE-GFP-LC3 was a generous gift from Prof. Dr. Noboru Mizushima (University of Tokio, Japan) [24]. pBABE-Puro and pBABE-Puro-HER2 plasmids were from Addgene (Addgene ID #1764 and #40978, respectively). pBicep-CMV-FLAG-BECN1 was already reported [25], pcDNA3.1-GRB2-HA was generated by conventional PCR cloning, and mutants GRB2(Y209A)-HA, GRB2(Y52A)-HA and GRB2(R86A-S88A)-HA were generated by VectorBuilder. pLKO.1 plasmid, pMD2G, psPAX2, were already reported [26], and pLKO.1-shRNA plasmids targeting *GRB2* (shGRB2#1, shGRB2#2 and shGRB2#3), were from Sigma’s Mission Library (TRCN0000029369, TRCN0000029371, TRCN0000029372) through Prof. Dr. Stefan Fröhling and Prof. Dr. Claudia Scholl (DKFZ, Heidelberg, Germany). HiBiT-GFP-LC3 was previously reported [27] and HiBiT-LC3 was from Promega (N2361) [17].

### Generation of stable cell lines

HeLa and HeLa-HiBiT-GFP stable cell lines expressing pcDNA 3.1, pcDNA 3.1-GRB2-WT-HA, pcDNA3.1-GRB2(Y52A)-HA, pcDNA3.1-GRB2(S86A-R88A)-HA, pcDNA3.1-GRB2(Y209A)-HA, as well as SKBR3-HiBiT-LC3 cells were generated after plasmid transfection followed by selection with Geneticin. Cells were maintained in media supplemented with the appropriate antibiotic. HeLa shGRB2 and SKBR3 shGRB2 knockdown stable cell lines were generated by lentiviral transduction according to an established protocol [26] and further selected with Puromycin.

BT-474-GRB2 CRISPR cells were generated using CRISPR technology as follows: Reagents and RNA guides (sgRNAs) were obtained from Synthego, and generation of BT-474-GRB2 CRISPR cell lines was developed according to manufacturer’s instructions. Cells were seeded in 6-well plates to reach 70% confluency the day of transfection. Ribonucleoprotein complexes (RNPs) were generated by diluting 5 μl of Lipofectamine Cas9 plus reagent with 20 pmol of each of the sgRNA, and 20 pmol of Cas9 nuclease in 125 μl of OPTI-MEM media. Upon 10 minutes incubation at room temperature, RNPs were mixed with the Lipofectamine CRISPRMAX reagent (7.5 μl) diluted in OPTI-MEM (125 μl) and incubated for 10 minutes. Mixture was added to the cells (containing 2 ml of full media per well) and incubated for two days. GRB2 depletion was analyzed by western blot.

### Transient siRNA transfection

GRB2 expression levels were downregulated by transfection of cells using three different Dicer-siRNA oligos (and corresponding non-targeting control). Transfection was done using HiPerfect transfection reagent (Qiagen) according to manufacturer’s instructions.

Oligos were purchased from Integrated DNA Technologies, including a negative control DsiRNA (51-01-14-04;CGUUAAUCGCGUAUAAUACGCGUAT), and three GRB2 oligos [hs.Ri.GRB2.13.1 (UGU AGA AUG CCA GAU UCC UUU UAU CAU), hs.Ri.GRB2.13.2 (CGA AGA AUG UGA UCA GAA CUG GUA C) and hs.Ri.GRB2.13.3 (AAC GAG GUA AUA UUU GAG GAA UCA A)].

### Transient simultaneous siRNA and DNA transfection

Simultaneous transfection of plasmid DNA and DsiRNA was performed using the TransIT-X2^®^ system from Mirus, according to manufacturer’s instructions.

### Cell lysates and protein quantification

Cells were washed twice in ice-cold PBS (Gibco) and lysed in ice-cold 50 mM Tris- HCl pH 7.4, 250 mM NaCl, 0.5% NP-40 containing protease (cOmplete EDTA-free, Roche) and phosphatase (PhosSTOP, Roche) inhibitors. After 10 minutes incubation at 4 °C lysates were cleared at 16.000 x *g* at 4 °C for 10 minutes. Protein was quantified using a protein stardard using Bovine serum albumin (BSA, Carl Roth) and using Bradford reagent (Bio-Rad). Cell debris was discarded, and supernatants were boiled in 4x Laemmli sample buffer (12% SDS, 30% β- mercaptoethanol, 60% Glycerol, 0.012% Bromophenol blue and 375 mM Tris pH 6.8) for 10 minutes at 95 °C for further analysis by Western blot. Protein extraction from CAM tumors was carried away as described in [28].

### Western blot analysis

Samples were analyzed by SDS-PAGE followed by western blot on a polyvinylidene difluoride (PVDF) membrane (VWR). Membranes were blocked in 5% low fat milk (Carl Roth) in TBS containing 0.1% Tween-20 (TBS-T) (blocking buffer) for 1 hour at room temperature. Membranes were then incubated in primary antibody diluted in antibody solution (3% BSA in TBST containing 0.05% sodium azide) overnight at 4 °C. Membranes were then washed with TBS-T (3 ×10 minutes) and incubated in horseradish peroxidase (HRP)-conjugated secondary antibody (1:5000) diluted in blocking buffer for 45 minutes. To avoid cross-reactivity of the heavy and light chains of the IgGs when analyzing immunoprecipitations, the Clean-Blot^TM^ IP detection reagent (Thermo Scientific) was used instead of the secondary antibody at a 1:400 dilution. Membranes were washed in 1x TBS-T (3 ×10 minutes), developed using ECL Prime solution (Fischer scientific), and imaged using FUSION software and imaging system. Uncropped western blots are provided within the Supplementary Data file.

### Co-immunoprecipitation

Cells were lysed in immunoprecipitation buffer (IP, 50 mM Tris- HCl pH 7.4, 150 mM NaCl, 0.5% NP-40) and pre-cleared with protein Protein A/G PLUS-Agarose beads (sc-2003; equilibrated 1:1 in IP buffer) for 1 hour at 4 °C. Beads were then taken away and antibody incubation was carried out overnight at 4 °C whilst rotating. Agarose beads were added the next morning for 1 hour. Flow through was removed and beads washed 3x in IP buffer and boiled 5 minutes at 95 °C in 4x Laemmli sample buffer.

### HiBiT-LC3 assay

The autophagy HiBiT-LC3 Reporter Assay was used to quantify autophagic flux through a bioluminescent reporter. Cells stably express a LC3 protein tagged to a HiBiT peptide, an 11 aminoacid peptide corresponding to the N-terminal luciferase subunit. Luminescence was detected upon addition of the detection mixture containing the Large BiT protein (LgBiT, C-terminal inactive luciferase subunit) and the luciferase substrate. Upon addition of this mixture, cells are lysed and the LgBiT binds to the HiBiT subunit with high affinity to generate an active luciferase enzyme that correlated with total LC3 reporter levels. Upon autophagy induction, cytosolic LC3-I is recruited to phagophores where it becomes conjugated to phosphatidylethanolamine, thereby forming LC3-II. Upon phagophore membrane elongation and closure to form the mature autophagosomal vesicle, a significant fraction of LC3 protein was captured within the lumen along with various cargo materials. When autolysosomes form, there was a degradation of both cargo material and the captured LC3 protein. Therefore, upon autophagy induction, less luminescent signal was detected.

SKBR3 and HeLa cells stably expressing either the HiBiT-LC3 or the GFP-HiBiT-LC3 were plated in white 96-well plates. The following day cells were treated for 3 hours with the corresponding treatment. After treatment, the lytic mixture containing the LgBiT protein (1:100) and the HiBiT substrate (1:50) diluted in the HiBiT lysis buffer was added at a 1:1 ratio with the media. Upon incubation for 10 minutes at room temperature, LC3 levels were determined through the luminescent signal normalized to cell number.

The HiBiT-LC3 reporter assay measured the autophagic flux through assessing the total LC3 levels. By contrast, the dual HiBiT-GFP-LC3 reporter allowed the assessment of autophagic flux by both, the autophagosome numbers through GFP-LC3 puncta analysis as well as of total LC3 levels using luminescence.

### Quantification of GFP-LC3 puncta assay

Cells were transfected using *Trans*IT^®^-LT1 (Mirus, MIR2300) reagent and fixed in 4% paraformaldehyde at room temperature for 10 min after treatments. Plates were washed twice with PBS followed by Hoechst staining at room temperature for 20 minutes, and GFP-LC3 puncta numbers of at least 50 cells per condition were monitored using a Zeiss AxioCam MRm, Carl Zeiss microscope.

### Assays for measuring PI3P-kinase activity

Analysis to ascertain the activity of VPS34, the catalytic subunit of the Class III PI3Kinase complex 1 (PI3KC3-C1) were performed using two assays:

EGFP-2xFYVE reporter: Cells were transfected with the corresponding plasmid and 24 h later treatments were performed. Cells were fixed in 4%PFA in PBS and EGFP-2xFYVE dots were determined in at least 50 cells per condition using a Zeiss AxioCam MRm, Carl Zeiss microscope.

In addition, an in vitro phosphatidylinositol 3-kinase assay was performed. Briefly, BECN1 was immunoprecipitated (see above) from the corresponding cells to isolate the components of the PI3KC3-C1. Immunoprecipitated complexes bound to the beads were washed 3x in IP buffer, 1x in TNE buffer (10 mM TrisHCl pH7.5, 100 mM NaCl, 1 mM EDTA), and further resuspended in 60 μl TNE buffer containing 6 μl ATP (0.5 mM in TNE) and 10 μl 100 mM MgCl_2_. Upon addition of the lipid substrate (0.1 mg/ml, V1711, Promega), the mixture was incubated for 20 minutes at room temperature. Kinase activity was measured on the beads supernatant using the ADP-Glo kinase assay kit (Promega) according to the manufacturer’s instructions.

### *In ovo* chick chorioallantoic membrane (CAM) assay

Freshly fertilized eggs were incubated at 38 °C in a 50% atmospheric humidity in a rotary incubator. On embryonic day 10, cells were implanted into the CAM membrane and tumors were extracted on day 17. Tumor identification was determined following morphological (unified mass formation differentiated from other membrane components) and angiogenesis features (increased vasculature developed surrounding the tumor).

Tumors were measured and cut in two pieces for both histological (4% PFA fixation) and western blot analysis (snap frozen in liquid nitrogen).

### Immunohistochemical staining

CAM tumors were fixed overnight in 4% PFA-PBS and further embedded in paraffin. Endogenous peroxidase was bleached by incubation on 3% H_2_O_2_ for 10 min followed by epitope demasking in 10 mM citrate buffer, pH 6. Samples were blocked in 2% normal goat serum (NGS) in PBS for 30 minutes, and primary antibody was diluted in 2% NGS and incubated overnight at 4 °C in a humid chamber. The next morning, two 5 min washes with PBS were done and incubation with the secondary antibody was performed for 2–4 hours at RT. Samples were washed twice for 5 minutes with PBS, and developed in freshly prepared developing solution (1% DAB in DAKO liquid DAB substrate). Quantification of p62 was achieved by stablishing a 0 to 4 score according to the percentage of p62 positive cells from a blind scorer. A minimum of 50 cells per sample were counted.

### Protein-protein docking

The structure coordinates of BECN1 ECD (PDB ID: 4DDP) and GRB2 domains (PDB IDs: 1JYR, 6SDF, and 2VWF) were imported from PDB database. The substrate peptides bound to crystal structures of GRB2 domains were deleted and all protein structures were minimized by steepest descent algorithm followed by conjugate gradient method. BECN1 ECD was docked into different GRB2 domains using ClusPro 2.0 server. The binding site residues of GRB2 SH2 and SH3 domains were specified as attraction sites. Similar weighted scores were obtained for the interaction of BECN1 with the three GRB2 domains: -966 (SH2), -928 (N-terminal SH3) and -961 (C-terminal SH3). However, it is not advisable to use these scores to rank binding modes. All molecular visualizations and images were generated using ChimeraX.

### Reverse phase protein array (RPPA)

The prospective clinical cohort [29] was used to print reverse phase protein arrays for targeted testing of protein expression was previously reported [23]. Briefly, protein lysates from snap fresh frozen breast cancer specimens were spotted on nitrocellulose-coated glass slides followed by blocking for 2 h at room temperature. Incubation with primary antibodies was performed overnight at 4 °C and binding was then detected with Alexa Fluor 680-conjugated secondary antibodies. Antibodies against GRB2 (Cell Signaling, #3972) and p62 (Progen, GP62-C) were tested for their use in RPPA as shown in Supplementary Fig. S6F, G. Representative slides were stained for total protein quantification using Fast Green FCF protein dye as described before [30], and signal intensities of individual spots were quantified using GenePixPro 7.0 (Molecular Services Inc.). Data pre-processing and quality control were performed using the RPPanalyzer R package [31].

### TCGA and METABRIC datasets analysis

Gene expression and associated clinical data for breast cancer patients were obtained from The Cancer Genome Atlas (TCGA) database (https://portal.gdc.cancer.gov/), as well as the Molecular Taxonomy of Breast Cancer International Consortium (METABRIC) and analyzed as previously described [32]. Briefly, *GRB2* expression was correlated with *SQSTM1* (p62) using the RNA-Seq Expectation–Maximization (RSEM) normalized gene expressions for the BRCA-TCGA patients and the Illumina HT-12 platform for the METABRIC patients, downloaded from the European Genome-Phenome Archive (http://www.ebi.ac.uk/ega) under accession number EGAS00000000083.

### Survival analysis of PiA-RPPA dataset

GRB2 and p62 protein expression levels from RPPA were split to the median. Overall Survival (OS) was computed by considering the patient’s date of death or the last follow-up date. Progression-Free Survival (PFS) was considered when local recurrences, distant metastasis or the patient’s death occurred. Kaplan–Meier survival curves and log-rank tests were computed with IBM SPSS Statistics 25 as described elsewhere [33].

### Statistical analysis

Statistical analysis was performed using the software Graph Pad Prism 7.0. Shown values represent mean values and standard error of the mean (SEM). Statistical significance was calculated using one-way ANOVA, two-way ANOVA or *t*-test. The degree of significance is set as significant *P* < 0.05 (*), highly significant *P* < 0.01 (**), strongly significant *P* < 0.001(***).

### Supplementary information

Supplementary data

## Data Availability

All data generated during the current study are available from the corresponding author on reasonable request.
