# Supplementary material for: GRB2 is a BECN1 interacting protein that regulates autophagy
Source: Cell Death Dis. 2024 Jan 5;15(1):14. doi: 10.1038/s41419-023-06387-7 (PMC10770341; doi:10.1038/s41419-023-06387-7)

# Supplementary information

## GRB2 is a BECN1 interacting protein that regulates autophagy

Jetsy-Karina Montero-Vergara[1], Kira Plachetta[1], Lisa Kinch[2], Stephan Bernhardt[3], Kriti Kashyap[4], Beth Levine[2, †], Lipi Thukral[4,5], Martina Vetter[5], Christoph Thomssen[5], Stefan Wiemann[3], Samuel Peña-Llopis[6,7], Verena Jendrossek[1], Silvia Vega-Rubin-de-Celis[1,*]

[1]Institute for Cell Biology, University Hospital Essen, Hufelandstrasse 55, D-45147 Essen, Germany

[2]University of Texas Southwestern Medical Center, 5323 Harry Hines Blvd., Dallas, TX, 75390, USA.

[3]Division of Molecular Genome Analysis, German Cancer Research Center (DKFZ), Im Neuenheimer Feld 280, D-69120 Heidelberg, Germany.

[4]CSIR-Institute of Genomics and Integrative Biology, Mathura Road, New Delhi, Delhi 110025, India.

[5]Academy of Scientific and Innovative Research (AcSIR), Ghaziabad 201002, India.

[6]Department of Gynaecology, Martin Luther University, Halle-Wittenberg, Ernst-Grube-Str. 40, D-06120 Halle (Saale), Germany.

[7]Translational Genomics. Department of Ophthalmology, University Hospital Essen, Essen, Germany.

[8]German Cancer Consortium (DKTK) and German Cancer Research Center (DKFZ), Heidelberg, Germany.

[†]Deceased.

[*]Corresponding author: Silvia.VegaRubindeCelis@UK-Essen.de

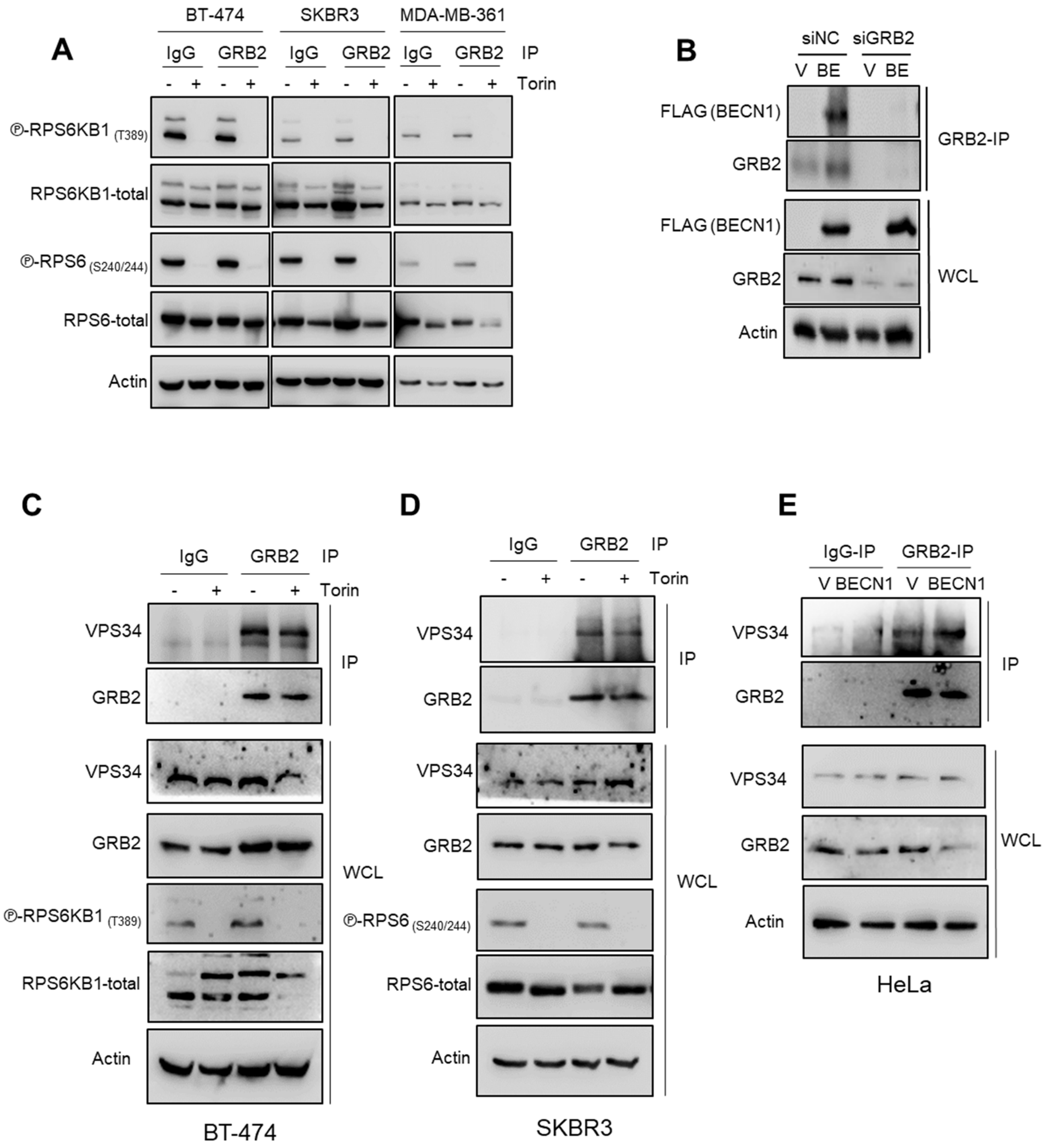


**Supplementary Figure S1. GRB2 co-immunoprecipitates with VPS34.** (A) Whole cell lysates from HER2 overexpressing cell lines were analyzed by WB with the indicated antibodies after treatment with Torin (250 nM, 3 h) or DMSO control. (B) HEK293T cells were transfected with FLAG-BECN1 (BE) or an empty vector control (V) and depleted of GRB2 using an siRNA oligo (siGRB2, or a non-targeting control, siNC). (C, D, E) Indicated cell lines were treated with Torin (250 nM, 3 h) and immunoprecipitated with a GRB2 antibody or an IgG control and analyzed by WB. WCL: Whole cell lysate.

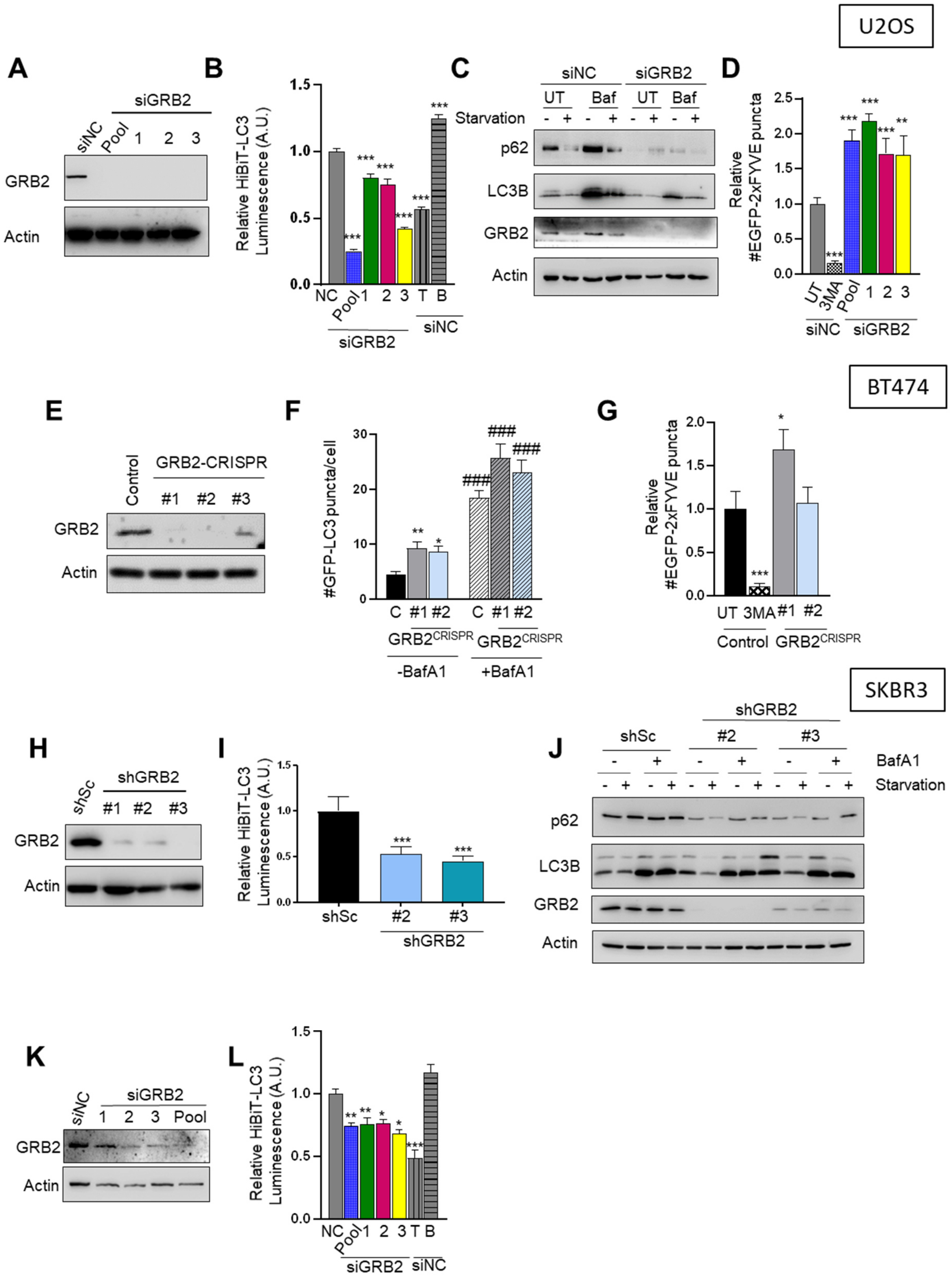


**Supplementary Figure S2. GRB2 depletion induces autophagy in multiple cell lines.** U2OS-HiBiT-LC3 cells were depleted of GRB2 using three individual siRNA or a Pool of all of them (or a non-targeting control, siNC) and analyzed by WB for assessment of total GRB2 levels (A), or autophagic flux by HiBiT-LC3 luminescence assay (B), WB (C), or EGFP-2xFYVE reporter assay (D). BT-474 CRISPR$^{GRB2}$ cells were analyzed by WT of total GRB2 levels (E), GFP-LC3 puncta assay (F), and or EGFP-2xFYVE reporter assay (G). SKBR3-HiBiT-LC3 cells with different shRNAs targeting *GRB2* (or an scrambled, non-targeting, control) were analyzed by WT of total GRB2 levels (H), HiBiT-LC3 luminescence (I), and WB (J). SKBR3-HiBiT-LC3 cells transiently transfected with the indicated siRNAs targeting *GRB2* (or a non-targeting control) were analyzed by WB (K), HiBiT-LC3 luminescence (L) and GFP-LC3 puncta assay (M). BafA1 (Bafilomycin A1, 100 nM, 3 h). Starvation is in HBSS for 3 h. Data are mean ± S.E.M. ***, $P < 0.001$; **, $P < 0.01$; *, $P < 0.05$, compared to untreated (UT) control, one-way ANOVA test.

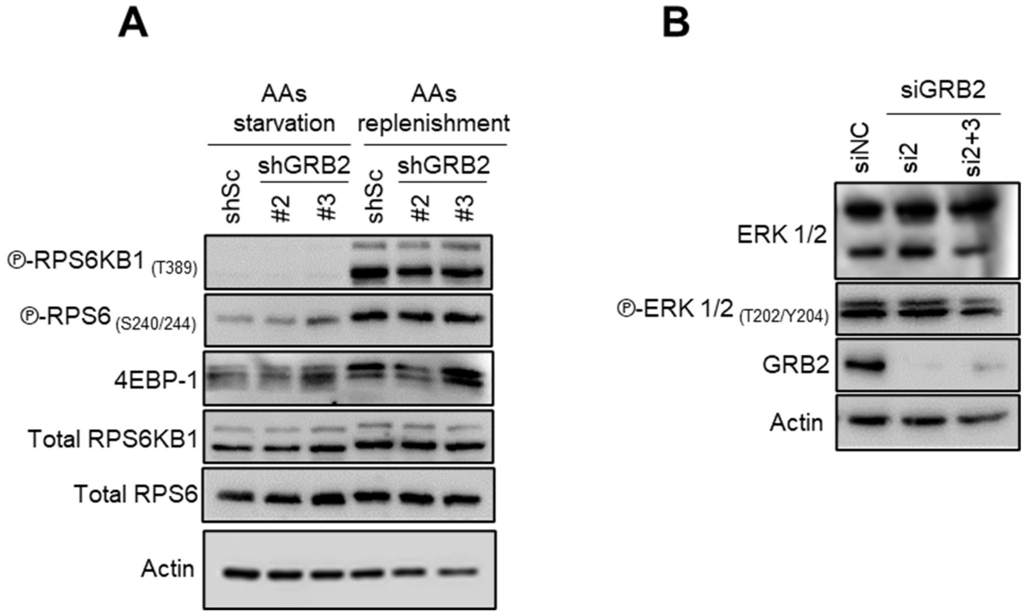


**Supplementary Figure S3. GRB2 depletion does not affect mTORC1 or MAPK pathways.** (A) Stable shGRB2 HeLa cells were analyzed by western blot after 50 min of amino acids (AAs) starvation or one hour of amino acids replenishment. (B) HeLa cells were transfected with the indicated siRNA oligos and analyzed by western blot. shSc: scrambled shRNA; #2, #3: shRNA targeting *GRB2*. siNC: non-targeting siRNA; si2 and si2+3: siRNAs targeting *GRB2* with one or a pool of oligos.

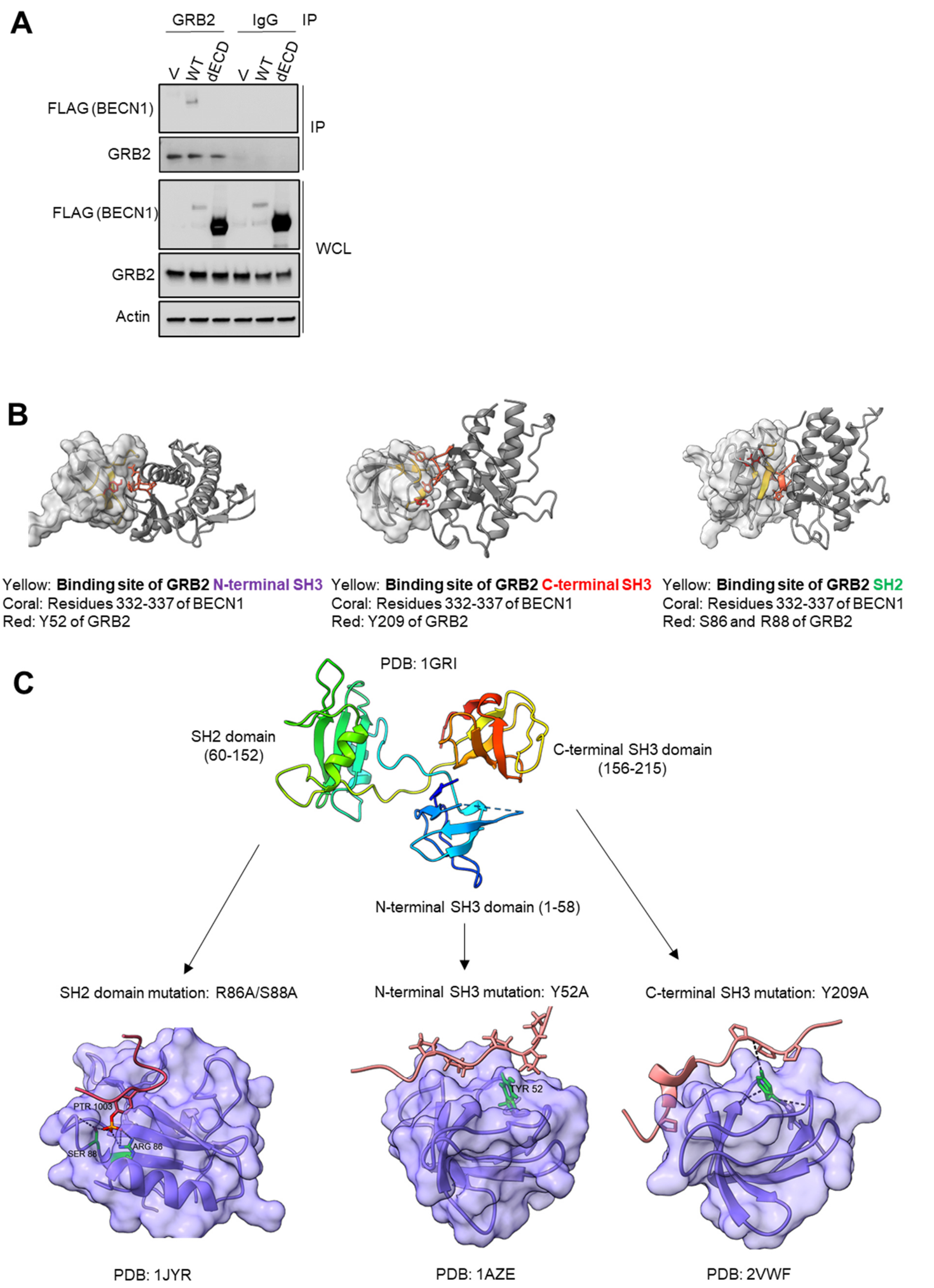


**Supplementary Figure S4. *In silico* modeling of GRB2 mutations.** (A) Immunoprecipitation (IP) analysis of HeLa cells transfected with the indicated FLAG-BECN1 constructs or an empty vector control (V). (B) Predicted binding modes of GRB2 regions with amino acids within the ECD domain of BECN1. (C) Selected mutations generated in this report.

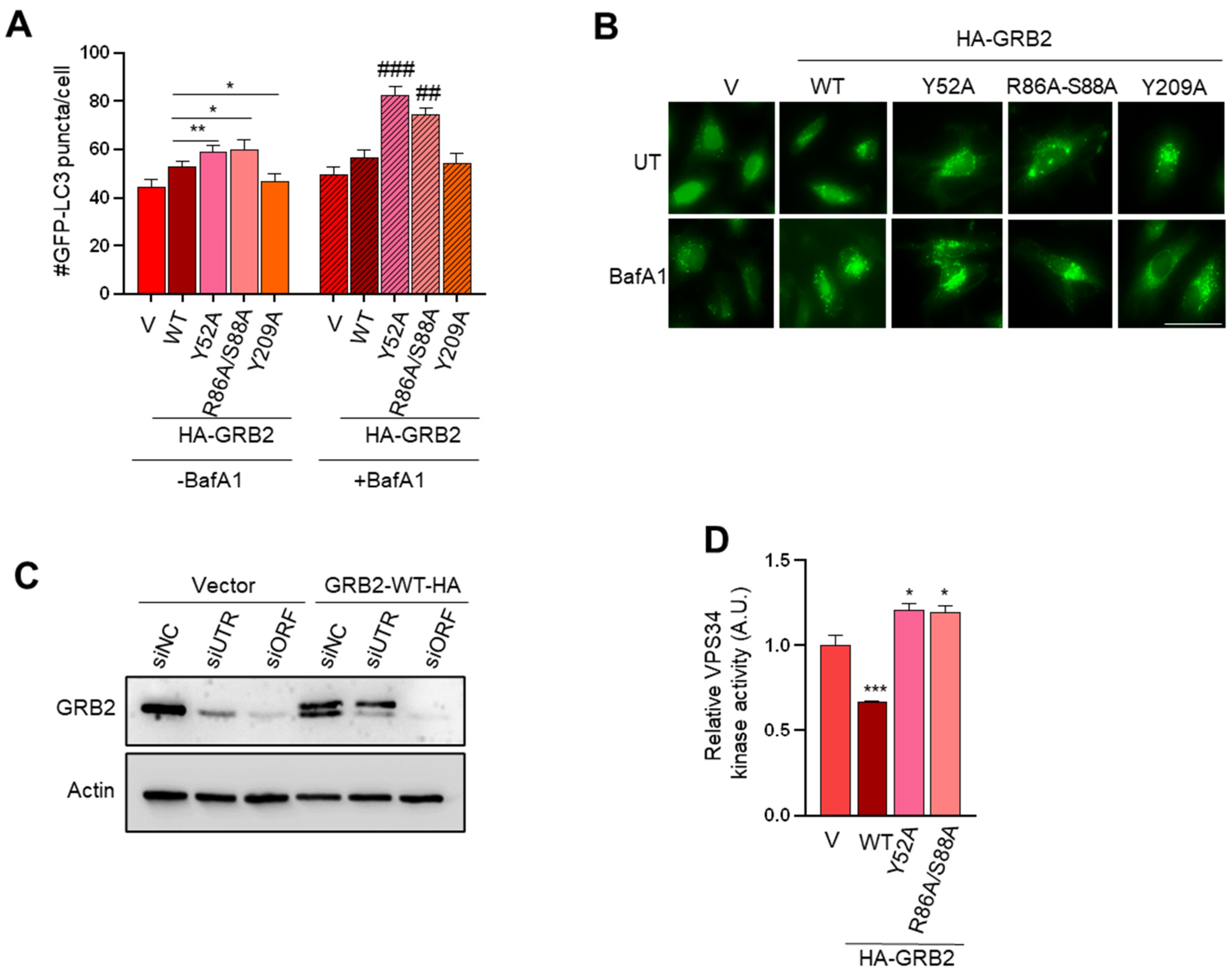


**Supplementary Figure S5. Analysis of GRB2 loss-of-function mutants.** (A, B) Numbers of autophagosomes in HeLa cells stably expressing the indicated GRB2 mutants were determined by the GFP-LC3 puncta formation assay (BafA1, 100 nM Bafilomycin A1, 3 h). (C) Western blot analysis of HeLa cells transfected with GRB2-WT-HA plasmid and siRNAs against the 3' UTR region of *GRB2* (siUTR) or its open reading frame (siORF) or a non-targeting control (siNC). (D) *In vitro* lipid kinase assay of VPS34 from HeLa cells expressing the indicated constructs (V: vector control; WT: GRB2-WT). ***, $P < 0.001$; **, $P < 0.01$; *, $P \leq 0.05$, one-way ANOVA test. # indicates comparison with its corresponding untreated sample. Scale bar 50 µm.

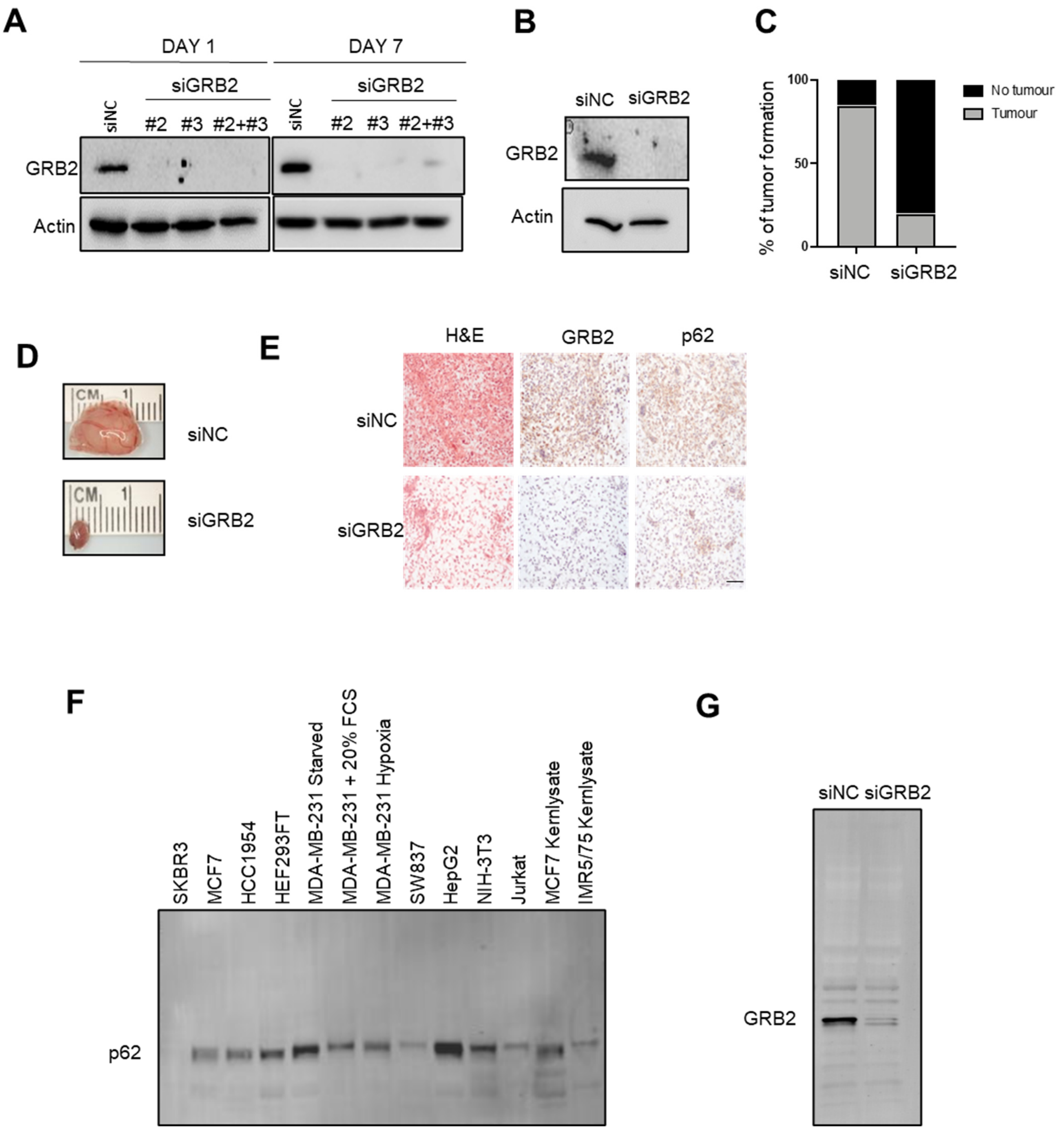


**Supplementary Figure S6. GRB2 depletion impairs tumor formation and growth in a CAM model.** (A) Western-blot testing analysis of *GRB2* knockdown in HeLa cells 1 or 7 days after transfection. (B) WB analysis of HeLa cells on the day of implantation. (C) Percentage (%) of tumor development (D) Representative pictures of extracted tumors and (E) histological analysis. NC: non-targeting control, siGRB2: *GRB2* siRNA. Western blot analysis of p62 (F) and GRB2 (G) antibodies used for the RPPA analysis. Scale bar 50 μm.

WB raw data

## Figure 1

1A-BECN1 (IP)

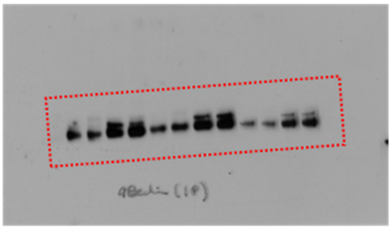

1A-BECN1/GRB2 (WCL)

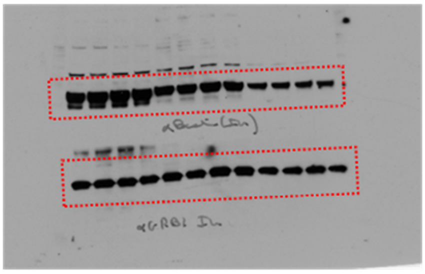

1A-GRB2 (IP)

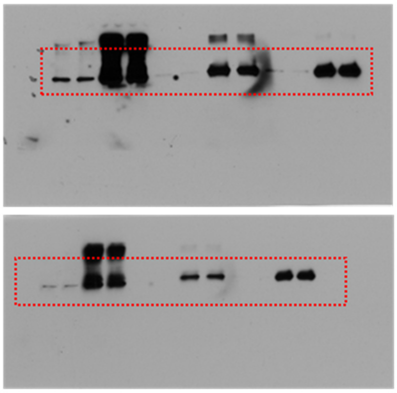

1A-tubulin (WCL)

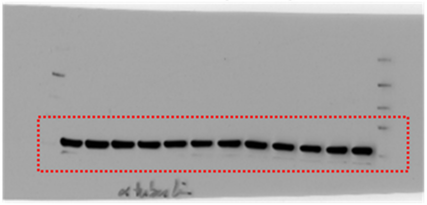

1B-FLAG (IP)

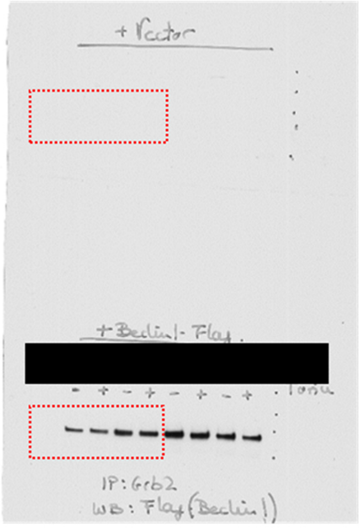


1B-FLAG (WCL)

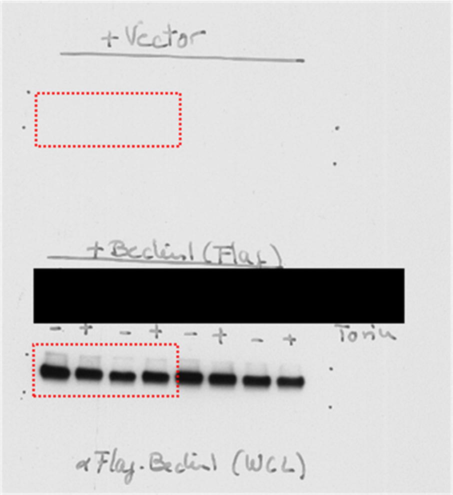


1B-Tubulin (WCL)

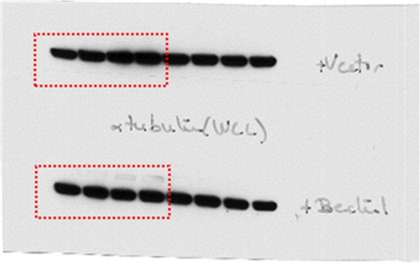

1B-HER2 (WCL)

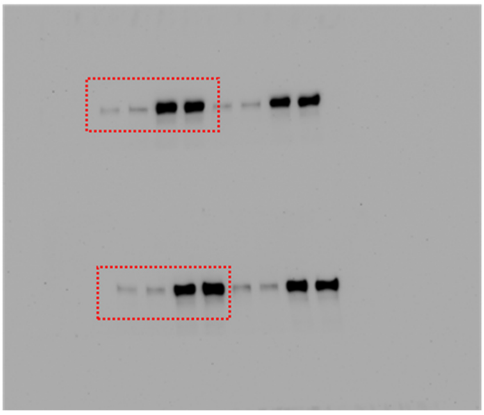

1B-GRB2 (WCL)

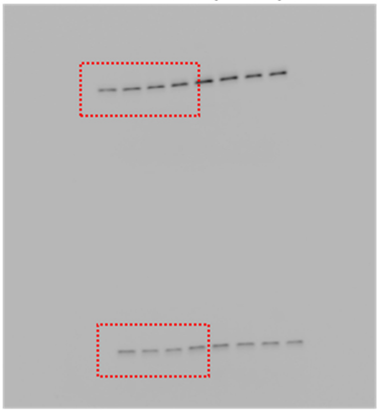

1B-GRB2 (IP)

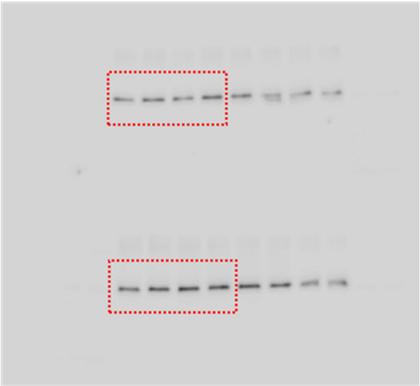

Figure 1

1C-BECN1

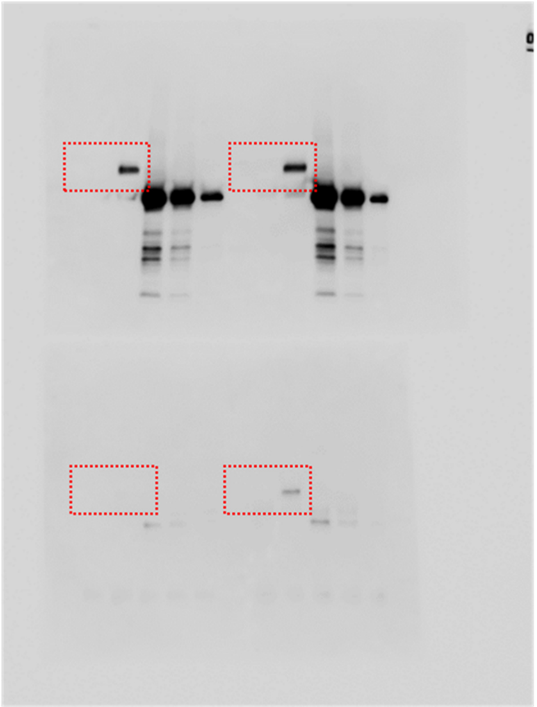

1C-GRB2

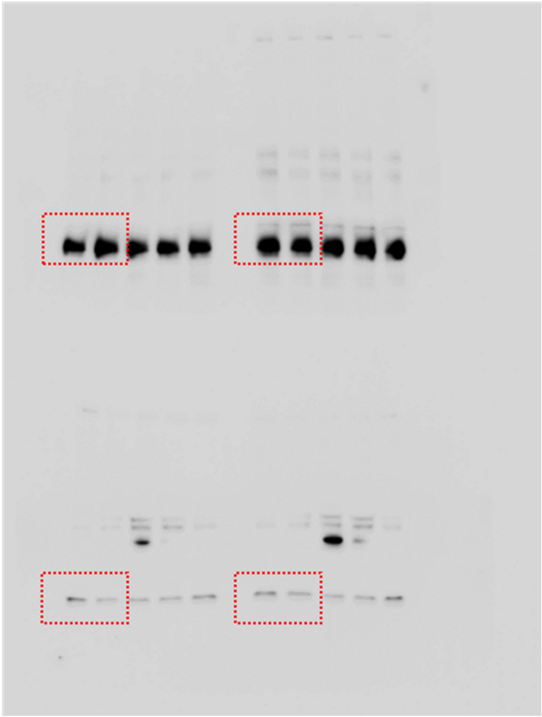

Figure 2

2A-GRB2

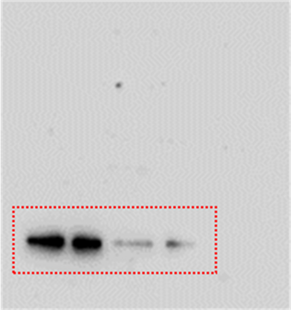

2A-actin

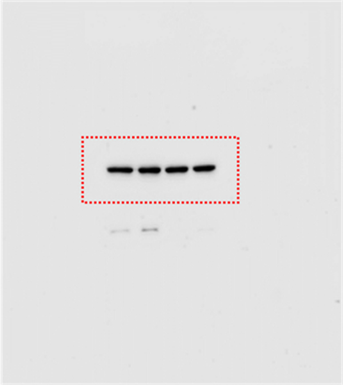

2B-p62

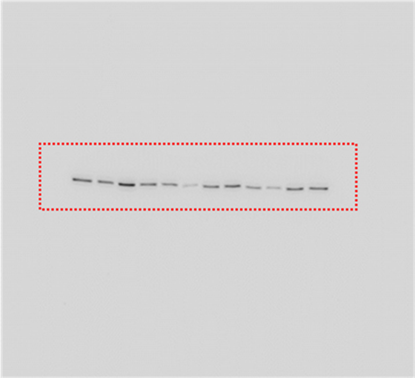

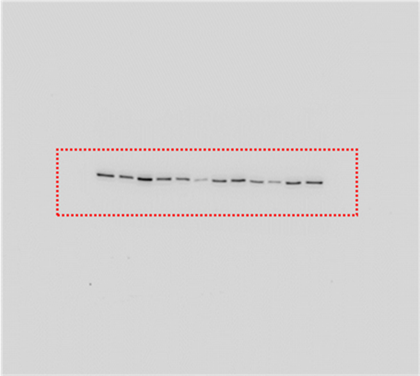

2B-LC3

2B-GRB2

2B-actin

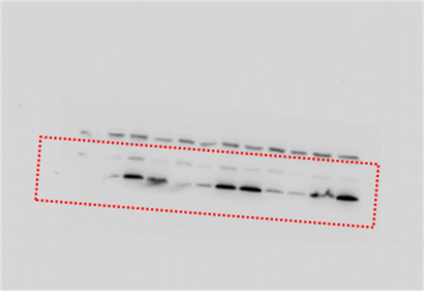

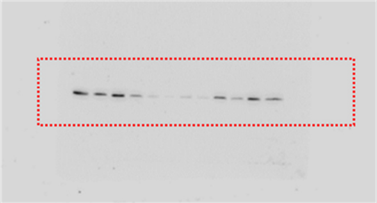

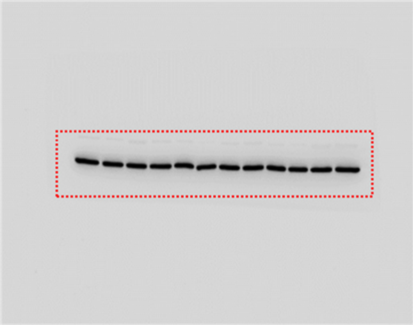

2J-GRB2

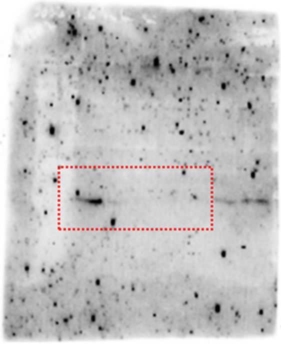

2J-actin

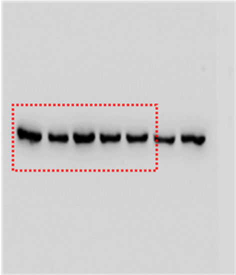

Figure 3

3C-GRB2

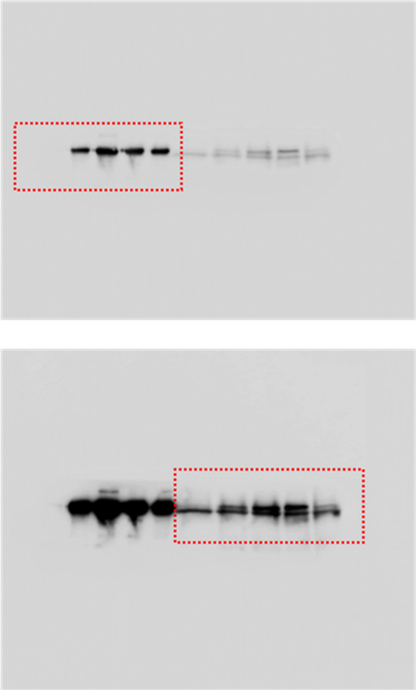

3C-BECN1

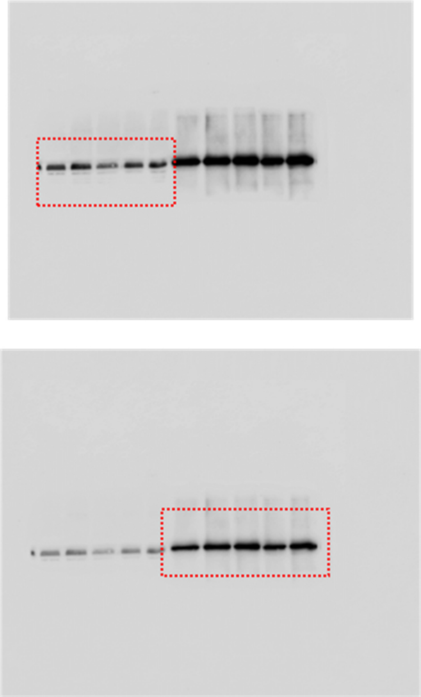

3C-actin

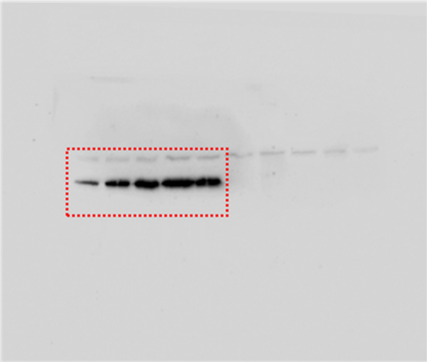

3D-HA(GRB2)

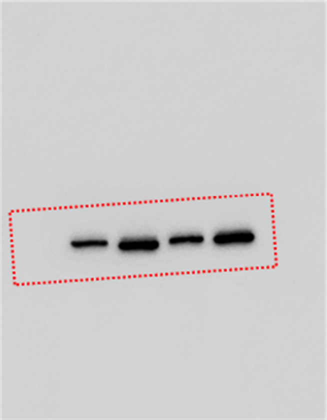

3D-actin

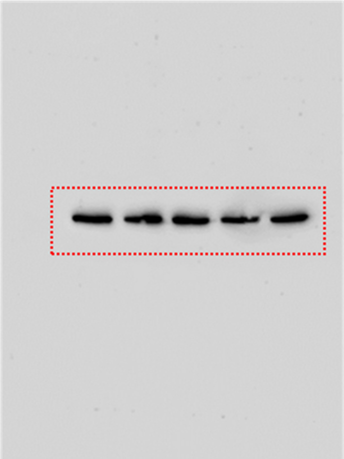

Figure 4

4A-GRB2

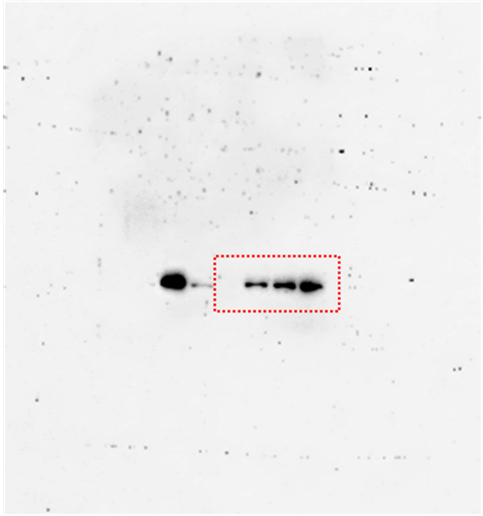

4A-actin

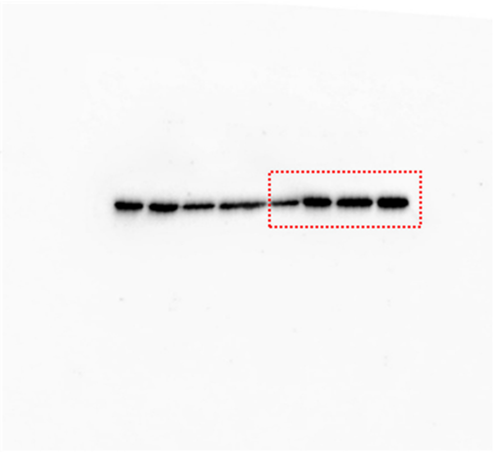

Supplementary Figure S1

S1A-pS6K1(T389)

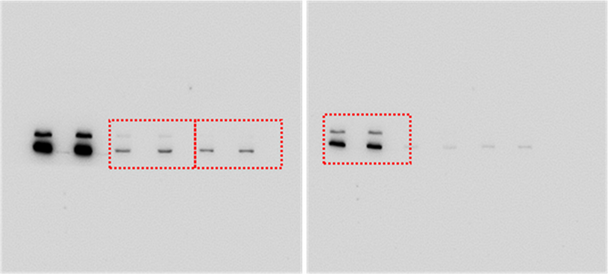

S1A-S6K1 total

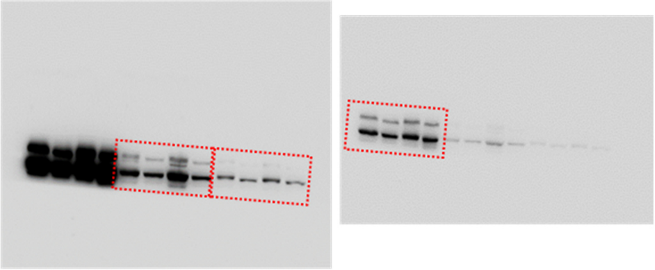

S1A-pS6(S240/244)

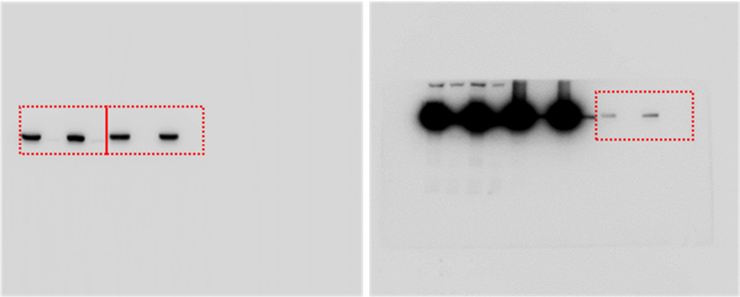

S1A-S6 total

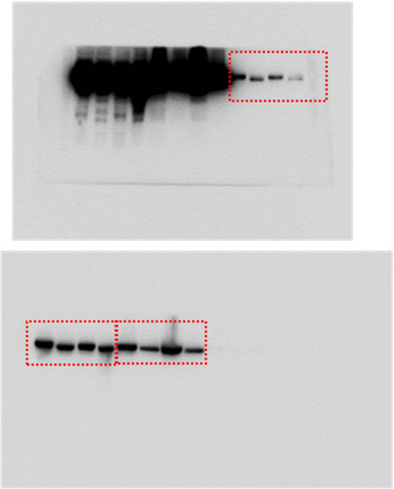

S1A-actin

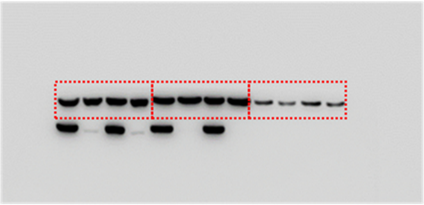

S1B-GRB2 (IP)

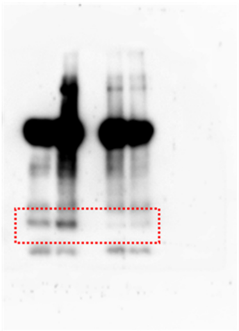

S1B-FLAG-BECN1 (IP)

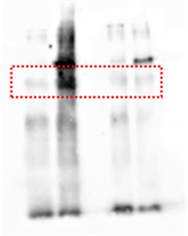

S1B-FLAG-BECN1 (top)
S1B-Actin (bottom)

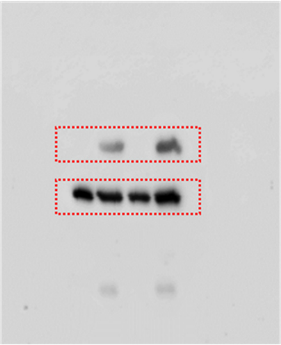

S1B-GRB2 (WCL)

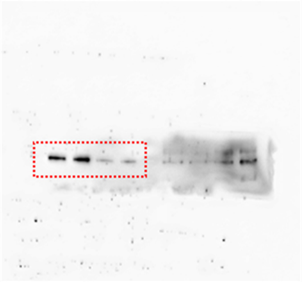

Supplementary Figure S1

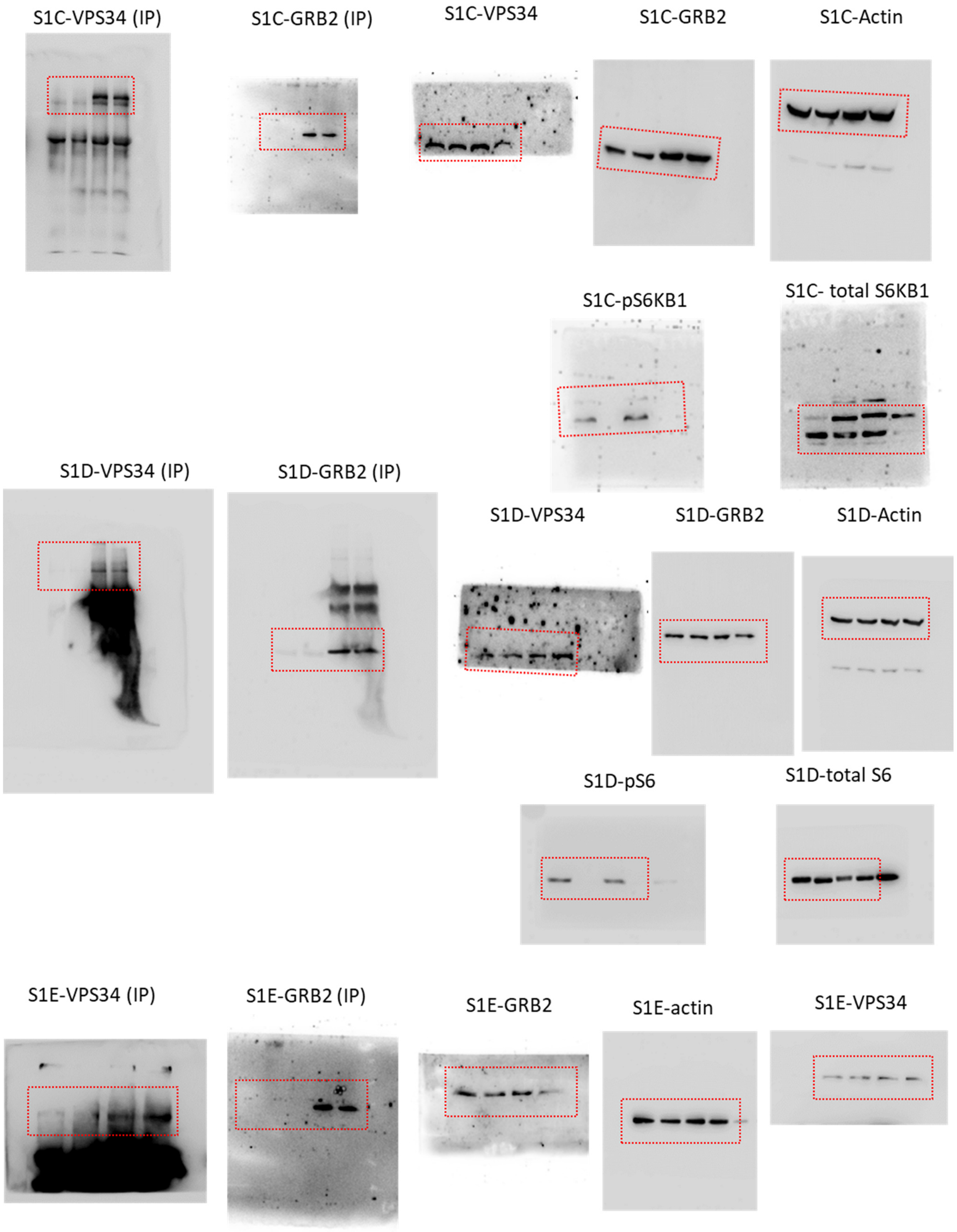

Supplementary Figure S2

S2A-GRB2

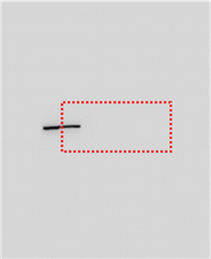

S2A-Actin

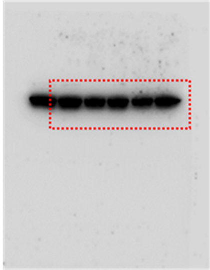

S2C-p62

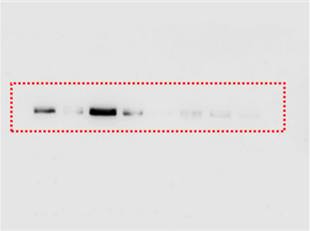

S2C-LC3

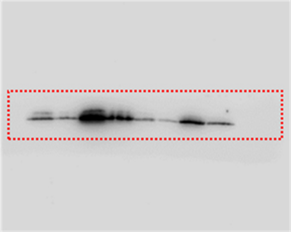

S2C-actin

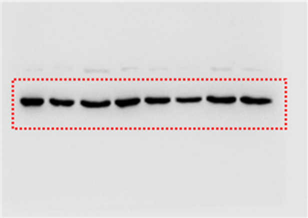

S2C-GRB2

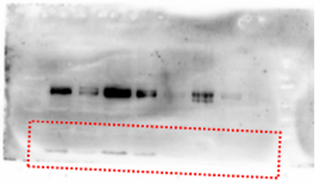

S2E-GRB2

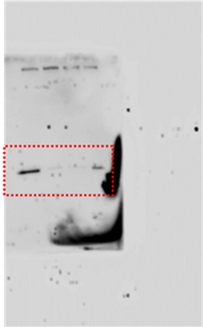

S2E-actin

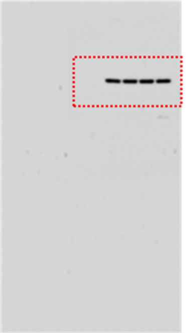

S2H-GRB2

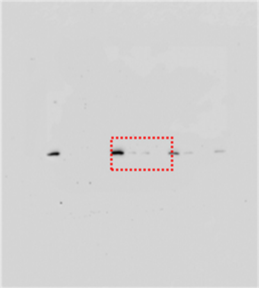

S2H-actin

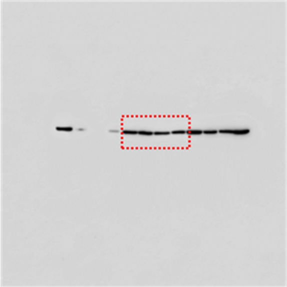

S2J-p62

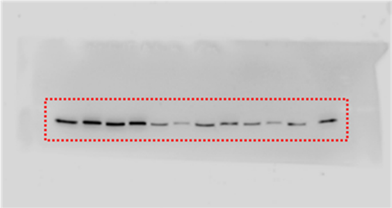

S2J-LC3

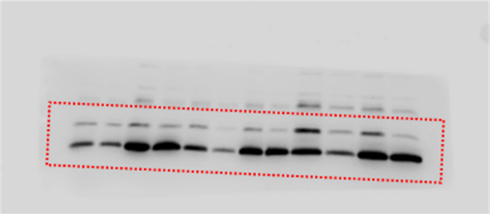

S2J-GRB2

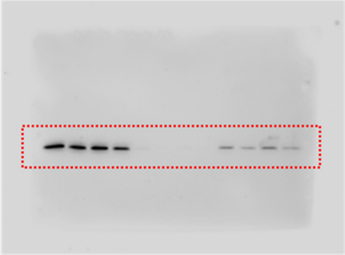

S2J-actin

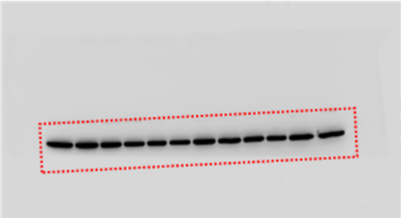

S2K-GRB2

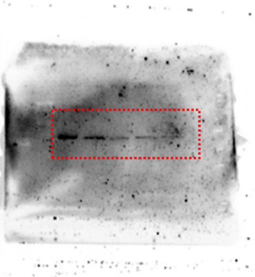

S2K-actin

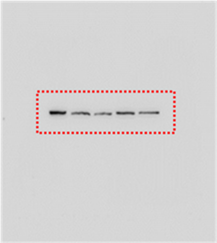

Supplementary Figure S3

S3A-pS6K1 (T389)

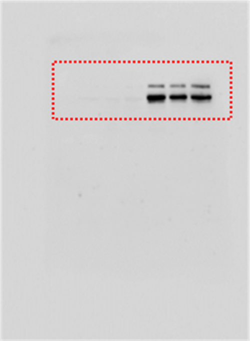

S3A-S6K1 (total)

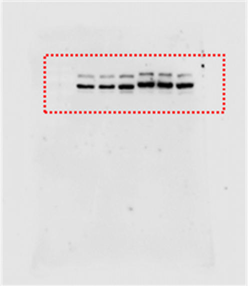

S3A-pS6 (S240/244)

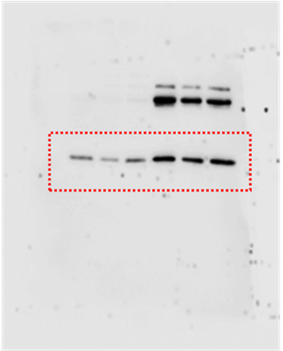

S3A-4EBP1

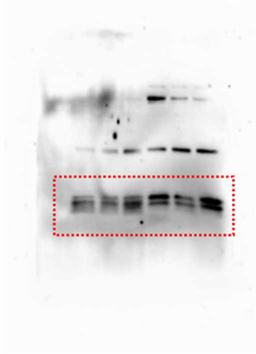

S3A-totalS6

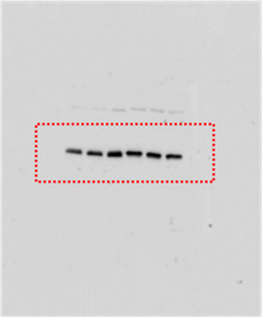

S3A-actin

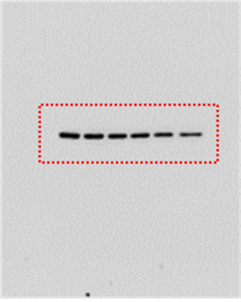

S3B-total ERK

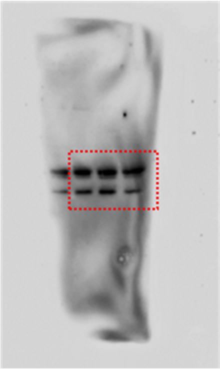

S3B-pERK

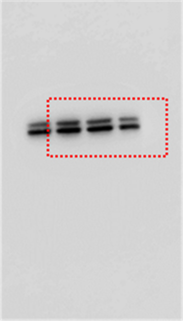

S3B-GRB2

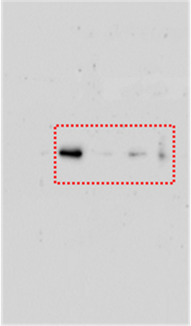

S3B-actin

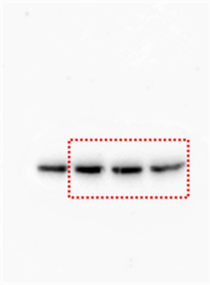

Supplementary Figure S4

S4A-FLAG (IP)

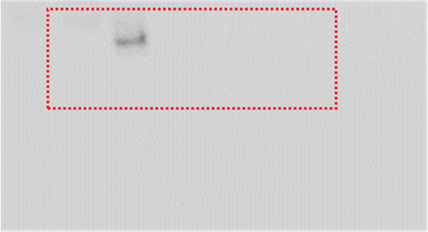

S4A-GRB2 (IP)

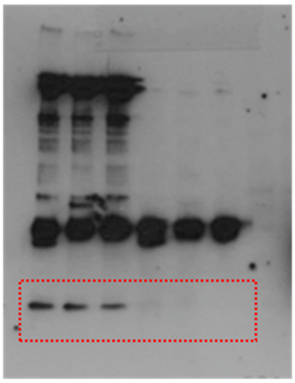

S4A-FLAG

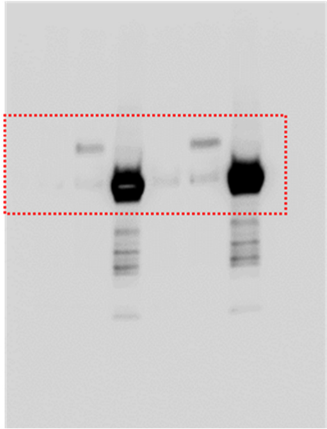

S4A-GRB2

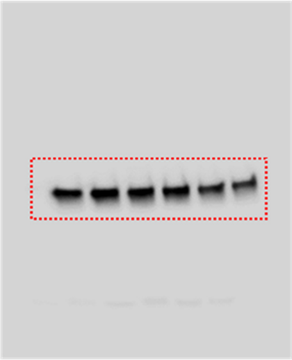

S4A-actin

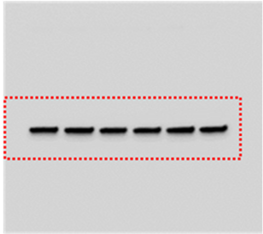

## Supplementary Figure S5

S5C-GRB2

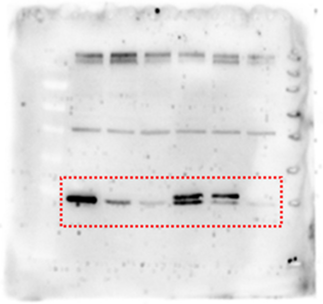

S5C-Actin

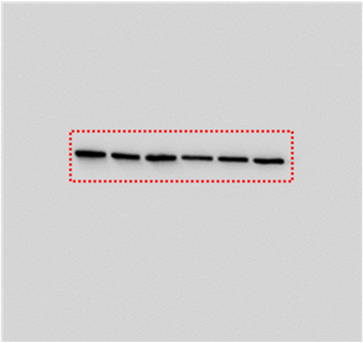

## Supplementary Figure S6

S6A-GRB2

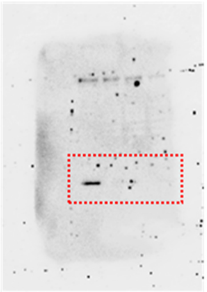

S6A-actin

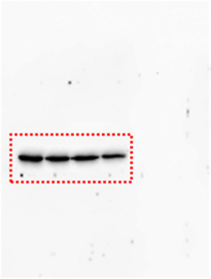

S6A-GRB2

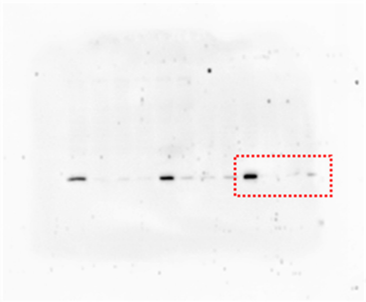

S6A-actin

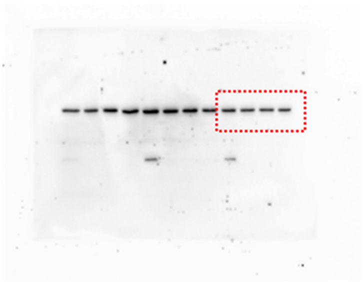

S6B-GRB2

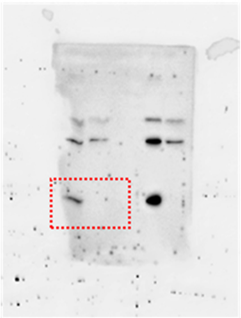

S6B-actin

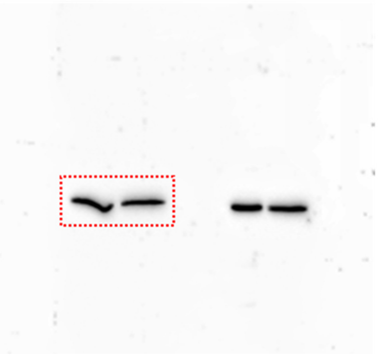

Supplement: Supplementary file 1 — Supplementary data [file 41419_2023_6387_MOESM1_ESM.pdf]
